# Enhancing cancer immunotherapy via inhibition of soluble epoxide hydrolase

**DOI:** 10.1073/pnas.2314085121

**Published:** 2024-02-08

**Authors:** Abigail G. Kelly, Weicang Wang, Eva Rothenberger, Jun Yang, Molly M. Gilligan, Franciele C. Kipper, Ahmed Attaya, Allison Gartung, Sung Hee Hwang, Michael J. Gillespie, Rachel L. Bayer, Katherine M. Quinlivan, Kimberly L. Torres, Sui Huang, Nicholas Mitsiades, Haixia Yang, Bruce D. Hammock, Dipak Panigrahy

**Affiliations:** ^a^Center for Vascular Biology Research, Beth Israel Deaconess Medical Center, Harvard Medical School, Boston, MA 02215; ^b^Department of Pathology, Beth Israel Deaconess Medical Center, Harvard Medical School, Boston, MA 02215; ^c^Cancer Center, Beth Israel Deaconess Medical Center, Harvard Medical School, Boston, MA 02215; ^d^Department of Entomology and Nematology, University of California, Davis, CA 95616; ^e^University of California Davis Comprehensive Cancer Center, Sacramento, CA 95817; ^f^Department of Food Science, Purdue University, West Lafayette, IN 47907; ^g^Institute of Systems Biology, Seattle, WA 98109; ^h^Department of Internal Medicine, University of California Davis, CA 95817; ^i^Department of Food Nutrition and Safety, College of Food Science and Nutritional Engineering, China Agricultural University, Beijing 100083, China

**Keywords:** immunonutrition, omega-3 fatty acids, eicosanoid, soluble epoxide hydrolase, inflammation resolution

## Abstract

Cancer therapy, including immunotherapy, is inherently limited by chronic inflammation-induced tumorigenesis and toxicity within the tumor microenvironment. Thus, stimulating the resolution of inflammation may enhance immunotherapy and improve the toxicity of immune checkpoint inhibition (ICI). As epoxy-fatty acids (EpFAs) are degraded by the enzyme soluble epoxide hydrolase (sEH), the inhibition of sEH increases endogenous EpFA levels to promote the resolution of cancer-associated inflammation. Here, we demonstrate that systemic treatment with ICI induces sEH expression in multiple murine cancer models. Dietary omega-3 polyunsaturated fatty acid supplementation and pharmacologic sEH inhibition, both alone and in combination, significantly enhance anti-tumor activity of ICI in these models. Notably, pharmacological abrogation of the sEH pathway alone or in combination with ICI counter-regulates an ICI-induced pro-inflammatory and pro-tumorigenic cytokine storm. Thus, modulating endogenous EpFA levels through dietary supplementation or sEH inhibition may represent a unique strategy to enhance the anti-tumor activity of paradigm cancer therapies.

## Significance

While immunotherapy is a front-line cancer treatment, unresolved chronic inflammation and toxicities often limit its anti-tumor activity. Endogenous clearance or resolution of inflammation may overcome this global intrinsic limitation of immunotherapy. Here, we demonstrate that immune checkpoint inhibitors (ICI) induce the expression of sEH (soluble epoxide hydrolase), which degrades the anti-inflammatory and pro-resolving EpFAs (epoxy-fatty acids) in the tumor microenvironment. Dietary ω-3 (omega-3) PUFAs (polyunsaturated fatty acids) supplementation and/or pharmacologic inhibition of sEH enhances ICI efficacy in multiple murine cancer models. Our results implicate the stabilization of endogenous EpFAs as a promising strategy in cancer therapy. Increasing endogenous anti-inflammatory and pro-resolving EpFAs via dietary supplementation and inhibition of sEH may be critically important as an adjuvant to conventional cancer therapies.

Cancer therapy, while designed to remove or kill tumor cells, can paradoxically stimulate tumor growth and resistance to treatment via pro-inflammatory response (1–6). The initiation and resolution of inflammation are active processes regulated by eicosanoids and pro-resolving lipid autacoid mediators, respectively (7). Cancer therapy, such as surgery and chemotherapy, may disrupt the resolution of inflammation (4, 5, 8). Unresolved inflammation in the tumor microenvironment (TME) can subsequently drive tumor progression and recurrence (4, 5, 9–11). Immune checkpoint inhibitors counter the endogenous inactivation or exhaustion of anti-tumor adaptive immunity by cytotoxic CD8^+^ T cells via blocking the programmed cell death (PD-1) or cytotoxic T lymphocyte-associated antigen-4 (CTLA4) pathways in various tumor types (12, 13). These immune-modulating agents have achieved durable remission in a subset of patients with melanoma and lung cancer (14). However, only 12.5% of patients respond to immune checkpoint inhibition (ICI) (15). The growing prevalence of immunotherapy-resistant malignancies has stimulated a flurry of clinical trials for combination therapies with other anti-cancer agents (e.g., chemotherapy) without significant benefits (16). Moreover, ICI is associated with various adverse inflammatory events (17, 18). ICI induces robust inflammation in the TME, which may lead to therapy failure and a dampened host immune system (19, 20). Thus, there is a critical unmet medical need for novel adjuvant cancer therapies that counteract therapy-associated inflammation and prevent therapy failure.

Omega-3 (ω-3) polyunsaturated fatty acids (PUFAs) are essential dietary precursors to the endogenous bioactive lipid mediators that regulate the resolution, or active clearance, of inflammation (21, 22). Alpha-linoleic acid, docosahexaenoic acid (DHA), and eicosapentaenoic acid (EPA) are the main dietary ω-3 PUFAs and are found in various nuts, seeds, and fish oils (23). ω-3 and ω-6 PUFAs are metabolized by cytochrome P450 enzymes (CYPs) to epoxy fatty acids (EpFAs), such as epoxydocosapentaenoic acids from DHA and epoxyeicosatetraenoic acids from EPA (24–26). EpFAs, including epoxyeicosatrienoic acids (EETs), regulate endothelial cell function, suppress pro-inflammatory cytokine production, reduce endoplasmic reticulum stress, and mediate inflammation resolution (27–31). ω-3 PUFAs have long been associated with both anti-inflammatory and anti-carcinogenic activity in animal disease models (32, 33). Importantly, EpFAs have not been well-characterized in the context of cancer or paradigm cancer therapies. Primary tumor growth (e.g., melanoma) is reduced in *Fat-1* transgenic mice which exhibits elevated endogenous ω-3 PUFAs levels (34). Randomized controlled trials have linked EPA and DHA dietary supplementation to a reduction in cardiovascular and cancer risk, though studies remain divided on the latter (35–37). Immuno-nutrition, the concept of modulating inflammation and immune response via nutrient consumption, for example, PUFAs (38), may improve survival in cancer patients undergoing cancer therapy (39).

EpFAs are rapidly metabolized to their corresponding diols, mainly by the enzyme soluble epoxide hydrolase (sEH) (40, 41). Pharmacological inhibition of sEH increases levels of EpFAs including EETs, which confer beneficial effects in various preclinical disease models (27, 42–53). Hydrolysis of EETs via sEH activity inhibits the production of pro-resolving mediators (54, 55). In contrast, sEH inhibition activates endogenous resolution pathways and stimulates the production of pro-resolving mediators, such as lipoxins and resolvins (53, 56). Recent clinical development of sEH inhibitors (sEHIs) has targeted various inflammation-associated diseases such as hypertension, diabetic neuropathy, and chronic obstructive pulmonary disease (COPD) (27, 41, 42, 57). Genetic deletion or pharmacological inhibition of sEH also prevents inflammation-induced carcinogenesis (46, 47, 58, 59). Dual sEH/COX2 inhibition counter-regulates a chemotherapy-induced cytokine storm and synergizes with front-line cancer therapies such as chemotherapy and enzalutamide to reduce tumor growth (9, 11, 60, 61). Inhibition of sEH reduces pancreatic tumor cell viability and enhances the anti-cancer activity of fenofibrate (62). Inhibition of sEH also potentiates the anti-tumor activity of ω-3 PUFAs in murine pancreatic ductal adenocarcinoma (63). sEH is a potential cancer biomarker, as it is elevated in at least 50% of individuals with hepatocellular cancer (64).

In this study, we evaluated whether the resolution of ICI-induced inflammation via sEH inhibition improves ICI efficacy using several murine cancer models. We found that treatment with immune checkpoint inhibitors, including anti-PD-1 or anti-CTLA-4, induced the expression of the sEH gene (*Ephx2*). We demonstrate that pharmacological blockade of sEH substantially enhanced the anti-tumor potency of immune checkpoint blockade in various preclinical cancer models. Dietary ω-3 PUFAs supplementation and pharmacologic sEH inhibition, both alone and in combination, significantly enhanced ICI efficacy in these models. While inflammatory marker expression in the TME increased after ICI treatment (e.g., *Il-1β, Tnfα*, *Ccl2*, and *Ccl4)*, the increase was significantly attenuated by a pharmacologic sEH inhibitor alone or in combination with ICIs. Pharmacologic sEH inhibition prevented a pro-inflammatory cytokine storm. Thus, modulating endogenous EpFA levels through dietary supplementation and sEH inhibition may represent a unique strategy to enhance the anti-tumor activity of cancer therapies.

## Results

### ICI Therapy Induces sEH Expression in Murine Cancer Models.

**Fig. 1.**
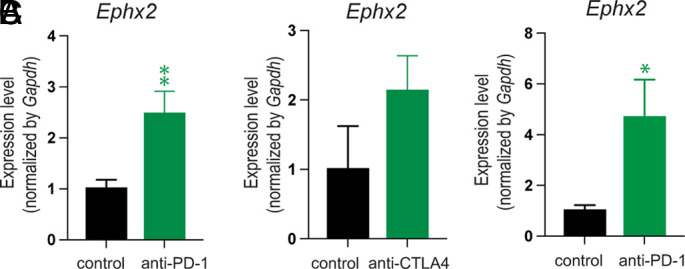
Immunotherapy induces sEH expression in murine tumor models. Expression of the genetic RNA transcript encoding sEH (*Ephx2*) in (*A* and *B*) MB49 tumor tissue from C57BL/6 mice and (*C*) RM1 tumor tissue from FAT-1 mice. Mice were subcutaneously inoculated with 1 × 10^5^ (RM1) or 1 × 10^6^ (MB49) tumor cells. Treatment was initiated with either anti-PD-1 (200 μg Q3D; “anti-PD-1”), anti-CTLA4 (200 μg first dose, then 100 μg Q3D; “anti-CTLA4”), or vehicle (“control”) once tumors reached ~200 mm^3^. n = 3 to 5 mice/group. Data are represented as mean ± SEM. *P < 0.05, **P < 0.01 versus control.

Because sEH is both a marker for and a cause of inflammation and tumorigenesis (10, 11, 65), we first measured gene expression of *Ephx2*, the gene that encodes sEH, in bladder and prostate tumors in mice treated with ICIs. C57BL/6 mice were subcutaneously inoculated with 1 × 10^6^ bladder cancer cells (MB49) and treated with systemic anti-PD-1 or vehicle once tumors were measured to be approximately 250 to 300 mm^3^. Tumor tissue harvested from anti-PD-1-treated mice exhibited significantly higher levels of *Ephx2* when compared to tumor tissue from vehicle-treated mice (Fig. 1*A*). To determine whether *Ephx2* gene induction was specific to ICI targeting PD-1, we utilized C57BL/6 mice inoculated with 1 × 10^6^ MB49 bladder tumor cells which were randomized to receive treatment with systemic anti-CTLA-4 or vehicle. Tumor tissues treated with anti-CTLA-4 also increased gene expression of sEH compared to vehicle-treated tumor tissue (Fig. 1*B*). We next evaluated immunotherapy-induced sEH expression in transgenic *Fat-1* mice, which are genetically engineered to produce high levels of endogenous ω-3 PUFAs (66). *Fat-1* mice were subcutaneously inoculated with 1 × 10^5^ prostate tumor cells (RM1), and established tumors (approximately 250 to 300 mm^3^) were treated with systemic anti-PD-1 or vehicle. Tumor tissues harvested from the anti-PD-1 treatment group exhibited a 2.5-fold increase in *Ephx2* expression (Fig. 1*C*). Here, we demonstrate that ICI can increase *Ephx2* expression, implicating a potential mechanism underlying immunotherapy failure and highlighting the need to further investigate sEH inhibitors as a novel adjuvant to improve the anti-tumor efficacy of ICI.

### Dietary ω-3 PUFAs Supplementation Inhibits Tumor Growth in Mice.

**Fig. 2.**
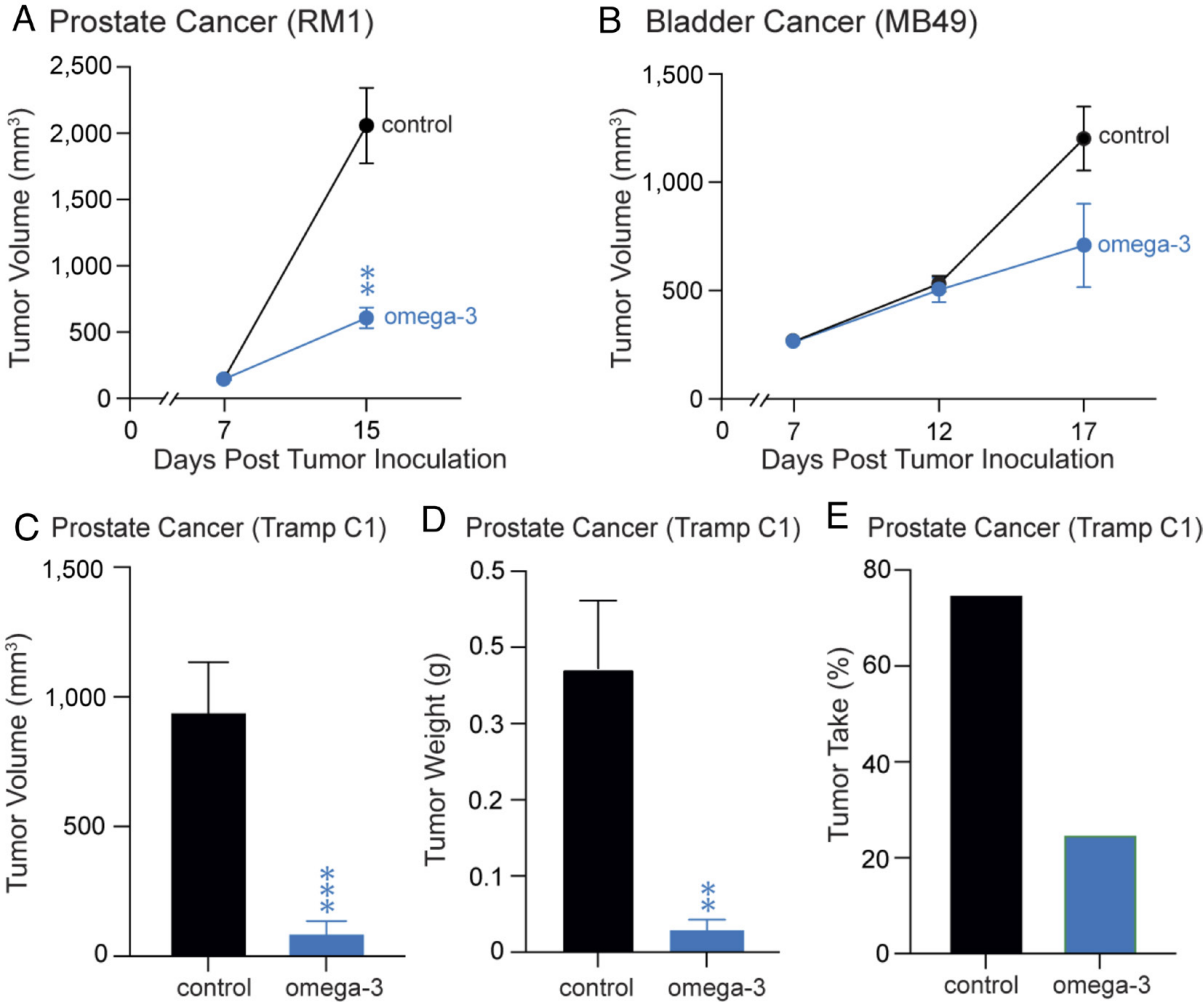
Dietary ω-3 PUFAs supplementation inhibits tumor growth in mice. (*A*) RM1 and (*B*) MB49 tumor growth in C57BL/6 mice. Mice were subcutaneously inoculated with 1 × 10^5^ (RM1) or 1 × 10^6^ (MB49) tumor cells following 12 d on an ω-3-enriched diet (AIN-93G with menhaden oil; “omega-3”) or a control diet (AIN-93G alone; “control”). n = 3 to 5 mice/group. Data are represented as mean ± SEM. **P < 0.01 versus control. Tramp C1 prostate tumor (*C*) volume and (*D*) weight on day 92 post-diet initiation, at which time mice were killed. Mice fed either a control diet (AIN-93G alone; “control”) or ω-3 PUFAs-enriched diet (AIN-93G with menhaden oil; “ω-3”). n = 20 mice per group. Data are represented as mean ± SEM. **P < 0.01, ***P < 0.001 versus control. (*E*) Prostate tumor take rate, defined as the presence of at least one palpable tumor, in Tramp C1 mice.

To investigate whether there is a potential therapeutic role for ω-3 PUFAs-derived EpFAs in cancer, we next characterized the potential anti-tumor activity of ω-3 PUFAs in murine tumor models. C57BL/6 mice were randomized to receive a control diet (*SI Appendix*, Table S1, AIN-93G) or an ω-3 PUFAs-enriched diet [increased ω-3/ω-6 ratio (ω-6 PUFAs deficient) diet] (*SI Appendix*, Table S2, AIN-93G and menhaden oil) for a total of 27 d (including 12 d pre-tumor implantation and 15 d post-inoculation). Mice were subcutaneously inoculated with 1 × 10^5^ RM1 prostate tumor cells after 12 d of diet administration. In this model, mice that received ω-3 PUFAs-enriched diet exhibited significantly less tumor growth at 15 d post-inoculation compared to mice fed the control diet (Fig. 2*A*). To determine whether this finding was limited to RM1 prostate tumors, we repeated the experiment using mice that were subcutaneously inoculated with 1 × 10^6^ MB49 bladder tumor cells and fed either the increased ω-3 PUFAs-enriched diet or control diet. At 17 d post-inoculation, the ω-3 PUFAs-enriched diet inhibited MB49 bladder tumor growth by approximately 40% (Fig. 2*B*). We next characterized the anti-tumor activity of dietary ω-3 PUFAs in a murine tumor model of prostate cancer using a transgenic cell line, transgenic adenocarcinoma of the prostate (TRAMP)-C1 cell line. Mice were subcutaneously inoculated with Tramp C1 tumor cells after 12 d of administration of either the increased ω-3/ω-6 ratio diet or control diet. By day 80 post-inoculation, the ω-3 PUFAs-enriched diet significantly decreased tumor volume and tumor weight per mouse when compared to the control group (Fig. 2 *C* and *D*). Moreover, mice fed the ω-3 PUFAs-enriched diet exhibited significantly less palpable prostate tumor growth (“tumor take”) with only 6 of 20 mice developing palpable tumors compared to the control group in which 17 of 20 mice developed palpable tumors (Fig. 2*E*). Thus, we demonstrate in both bladder and prostate cancer models that dietary ω-3 PUFAs supplementation significantly inhibited tumor growth, suggesting that endogenous lipid metabolites derived from dietary ω-3 PUFAs may play a critical inhibitory role in carcinogenesis.

### Dietary ω-3 PUFAs Supplementation Enhances ICI Efficacy In Vivo.

**Fig. 3.**
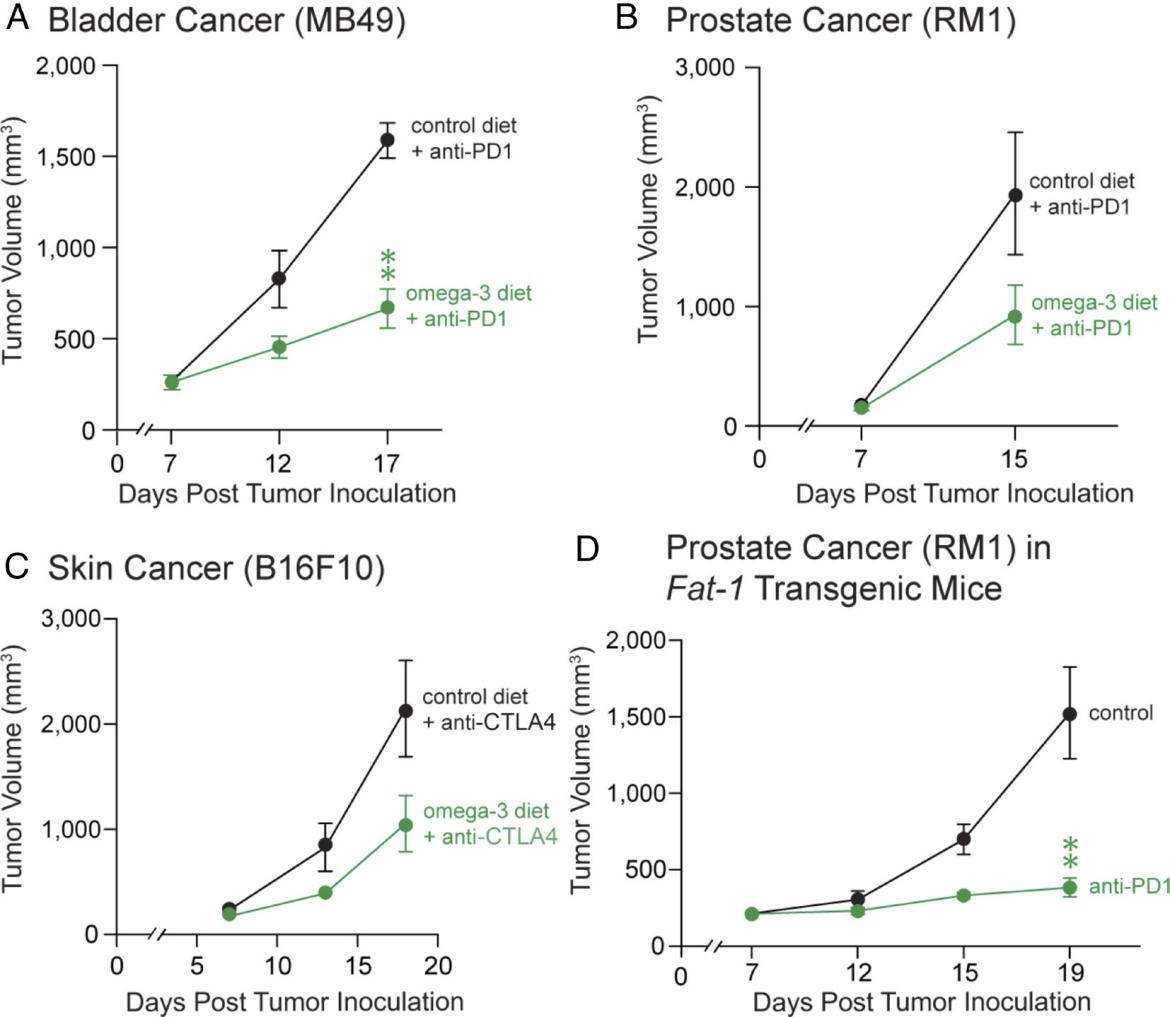
Dietary ω-3 supplementation in combination with ICI inhibits tumor growth. (*A*) MB49 tumor growth, (*B*) RM1 tumor growth, and (*C*) B16F10 tumor growth in C57BL/6 mice. Mice were subcutaneously inoculated with 1 × 10^5^ (RM1) or 1 × 10^6^ (MB49, B16F10) tumor cells following 12 d on an ω-3-rich diet or control diet. Treatment was initiated with either anti-PD1 (200 μg Q3D), anti-CTLA4 (200 μg first dose, then 100 μg Q3D), or vehicle (“control”) once tumors reached ~200 mm^3^. n = 3 to 5 mice per group. Data are represented as mean ± SEM. **P < 0.01 versus control. (*D*) RM1 tumor growth in *Fat-1* transgenic mice. Mice were subcutaneously inoculated with 5 × 10^4^ tumor cells and treatment was initiated with either anti-PD1 (200 μg Q3D) or no treatment (control) once tumors reached ~200 mm^3^. n = 3 to 5 mice/group. Data are represented as mean ± SEM. **P < 0.01 versus control.

We next investigated whether dietary ω-3 PUFAs supplementation improves the efficacy of immunotherapy. C57BL/6 mice were administered either ω-3 PUFAs-enriched diet or a control diet for 12 d, at which point they were subcutaneously inoculated with either 1 × 10^6^ MB49 bladder or 1 × 10^5^ RM1 prostate tumor cells. All mice were then treated with systemic anti-PD-1 therapy. By day 15 (RM1 prostate) or day 17 (MB49 bladder) post-inoculation, tumor-bearing mice were treated with anti-PD-1 and fed the increased ω-3/ω-6 ratio diet exhibited less tumor growth than mice that were treated with anti-PD-1 and fed the control diet (Fig. 3 *A* and *B*). To determine whether the observed enhancement of ICI with dietary ω-3 PUFAs was limited to MB49 bladder and RM1 prostate tumors, we utilized melanoma tumor cells (B16F10). Mice were administered either the increased ω-3/ω-6 ratio diet or a control diet for 12 d, at which point they were inoculated with B16F10 tumor cells and subsequently treated with systemic anti-CTLA4 once tumors were established. In this tumor model, concurrent treatment with anti-CTLA4 and dietary ω-3 PUFAs supplementation resulted in an approximately 35% inhibition of tumor growth in mice, compared to treatment with anti-CTLA4 and the control diet (Fig. 3*C*). To support that increased endogenous ω-3 PUFAs/ ω-6 PUFAs ratio enhances ICI efficacy in our tumor models, we utilized *Fat-1* mice (66). The utilization of transgenic *Fat-1* mice with an elevated tissue expression of ω-3 fatty acids removes any confounding factors associated with different dietary treatments (67). *Fat-1* mice were fed the control diet, subcutaneously inoculated with 5 × 10^4^ RM1 prostate tumor cells, and subsequently treated with systemic anti-PD-1. Treatment with anti-PD-1 drastically inhibited tumor growth in *Fat-1* mice at day 19 post-tumor cell inoculation (Fig. 3*D*). Taken together, these studies demonstrate that either dietary or genetic enrichment of endogenous ω-3 PUFAs improves ICI efficacy in multiple murine tumor models.

### Pharmacologic sEH Inhibition Enhances ICI Efficacy In Vivo.

**Fig. 4.**
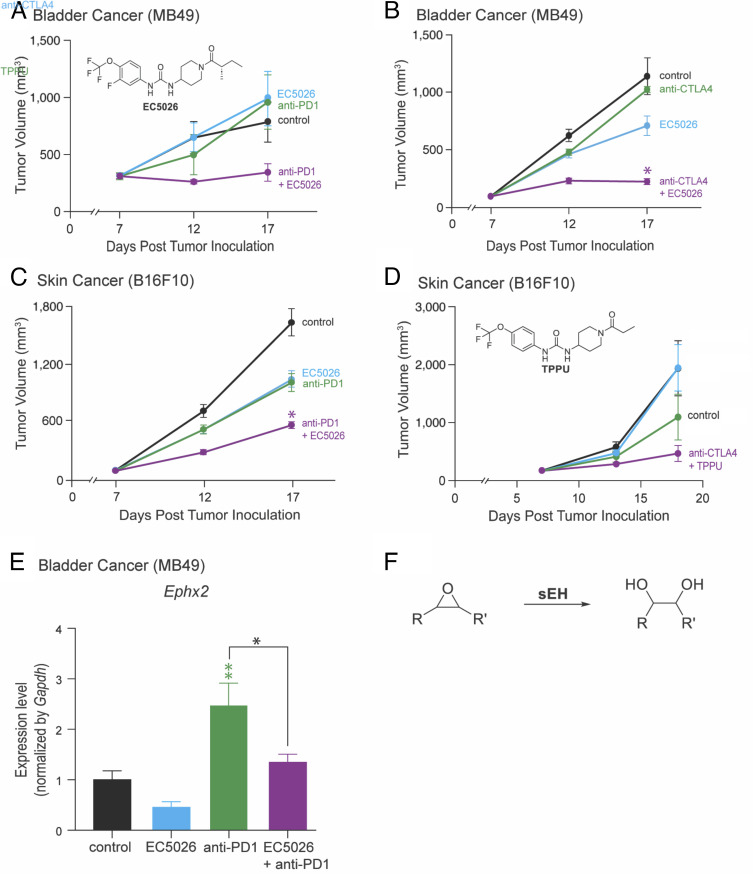
sEH inhibition enhances ICI inhibition of tumor growth in mice on high omega-6 diet. (*A* and *B*) MB49 tumor growth in C57BL/6 mice. Mice were subcutaneously inoculated with 1 × 10^6^ tumor cells following 12 d on a high omega-6 diet (AIN-76A). Treatment was initiated with anti-PD1 (200 μg Q3D) or anti-CTLA4 (200 μg first dose, then 100 μg Q3D), EC5026 (5 mg/kg/day), a combination of either anti-PD1 or anti-CTLA4 with EC5026, or vehicle (“control”) once tumors reached ~200 mm^3^. n = 3 to 5 mice per group for (*A*) and n = 8 to 10 mice for (*B*). *P < 0.05 versus control. (*C* and *D*) B16F10 tumor growth in C57BL/6 mice. Mice were subcutaneously inoculated with 1 × 10^6^ tumor cells following 12 d on a high omega-6 diet (AIN-76A). Treatment was initiated with anti-PD1 (200 μg Q3D) or anti-CTLA4 (200 μg first dose, then 100 μg Q3D), EC5026 (5 mg/kg/day) or TPPU (5 mg/kg/day), a combination of either anti-PD1/EC5026 or anti-CTLA4/TPPU, or vehicle (“control”) once tumors reached ~200 mm^3^. n = 8 to 10 mice per group for (*C*) and n = 4 to 5 mice per group for (*D*). Data are represented as mean ± SEM. *P < 0.05 versus control. (*E*) Expression of the gene encoding sEH (*Ephx2*) in tumor tissues harvested from mice utilized in (*A*), as described above. Data are represented as mean ± SEM. *P < 0.05, **P < 0.01, ***P < 0.001 versus control (no bracket) or comparing two groups (indicated by bracket). (*F*) The catalytic reaction formula for sEH.

ICI induces the gene expression of sEH and enrichment of endogenous ω-3 PUFAs, the precursors of ω-3 EpFAs, and enhances ICI efficacy in murine tumor models (Figs. 2 and 3). Therefore, we next determined whether pharmacologic inhibition of sEH could enhance ICI efficacy in our tumor models. C57BL/6 mice were subcutaneously inoculated with 1 × 10^6^ MB49 tumor cells and randomized into the following treatment groups: systemic ICI (anti-PD-1), vehicle, systemic sEHI (EC5026), or a combination of systemic ICI and EC5026. Treatment was initiated once MB49 bladder tumors reached a tumor volume of approximately 300 to 315 mm^3^. While mice treated with either anti-PD-1 alone (200 µg Q3 days) or EC5026 alone did not exhibit decreased tumor growth compared to the control group, mice treated with a combination of anti-PD-1 and EC5026 showed reduced tumor growth compared to all other groups (Fig. 4*A*). We repeated this experiment initiating treatment with anti-CTLA4 when MB49 bladder tumors reached a tumor volume of approximately 100 to 200 mm^3^. While mice treated with anti-CTLA4 alone did not exhibit significantly decreased tumor growth compared to the control group, the combination of EC5026 and anti-CTLA4 treatment significantly suppressed MB49 bladder cancer tumor growth compared to control (Fig. 4*B*). To determine whether this activity was limited to the specific tumor cell type used, we utilized melanoma (B16F10) tumor cells. Mice were subcutaneously inoculated with 1 × 10^6^ B16F10 tumor cells and randomized into the following treatment groups: systemic ICI (anti-PD-1), vehicle, systemic EC5026, or a combination of systemic anti-PD-1 and EC5026. Similar to our previous findings, the combination of anti-PD-1 and EC5026 significantly inhibited primary tumor growth via additive anti-tumor activity compared to monotherapy or the control group (Fig. 4*C*). To show that these findings were not unique to EC5026, we next utilized a chemically related sEHI TPPU (68). Mice were subcutaneously inoculated with 1 × 10^6^ B16F10 tumor cells and randomized into the following systemic treatment groups: vehicle, anti-CTLA-4, TPPU, or a combination of anti-CTLA-4 and TPPU. Accordingly, tumor growth was drastically suppressed in mice treated with a combination of anti-CTLA-4 and TPPU compared to mice treated with vehicle, anti-CTLA-4, or TPPU alone (Fig. 4*D*). Thus, our results demonstrate that sEHIs enhance ICI in multiple murine tumor models, regardless of the specific ICI or sEHI used.

After demonstrating that ICI increases gene expression of sEH in vivo (Fig. 1), we next investigated whether treatment with sEHI could appreciably counter this effect in our tumor models. MB49 tumors from mice treated with either anti-PD-1 alone, vehicle, EC5026 alone, or the combination of anti-PD-1 and EC5026 (Fig. 4*A*) were harvested and *Ephx2* expression was quantified. The combined treatment of ICI with sEHI reduced *Ephx2* expression compared to treatment with ICI alone (Fig. 4*E*). We further demonstrated that tumors from mice treated with both anti-PD-1 and EC5026 did not exhibit increased *Ephx2* expression, supporting that pharmacologic sEH inhibition counters ICI-induced *Ephx2* expression (Fig. 4 *E* and *F*).

### Pharmacologic sEH Inhibition Counter-Regulates Pro-Inflammatory Cytokine Storm and Increases Cytotoxic CD8^+^ T Cells.

**Fig. 5.**
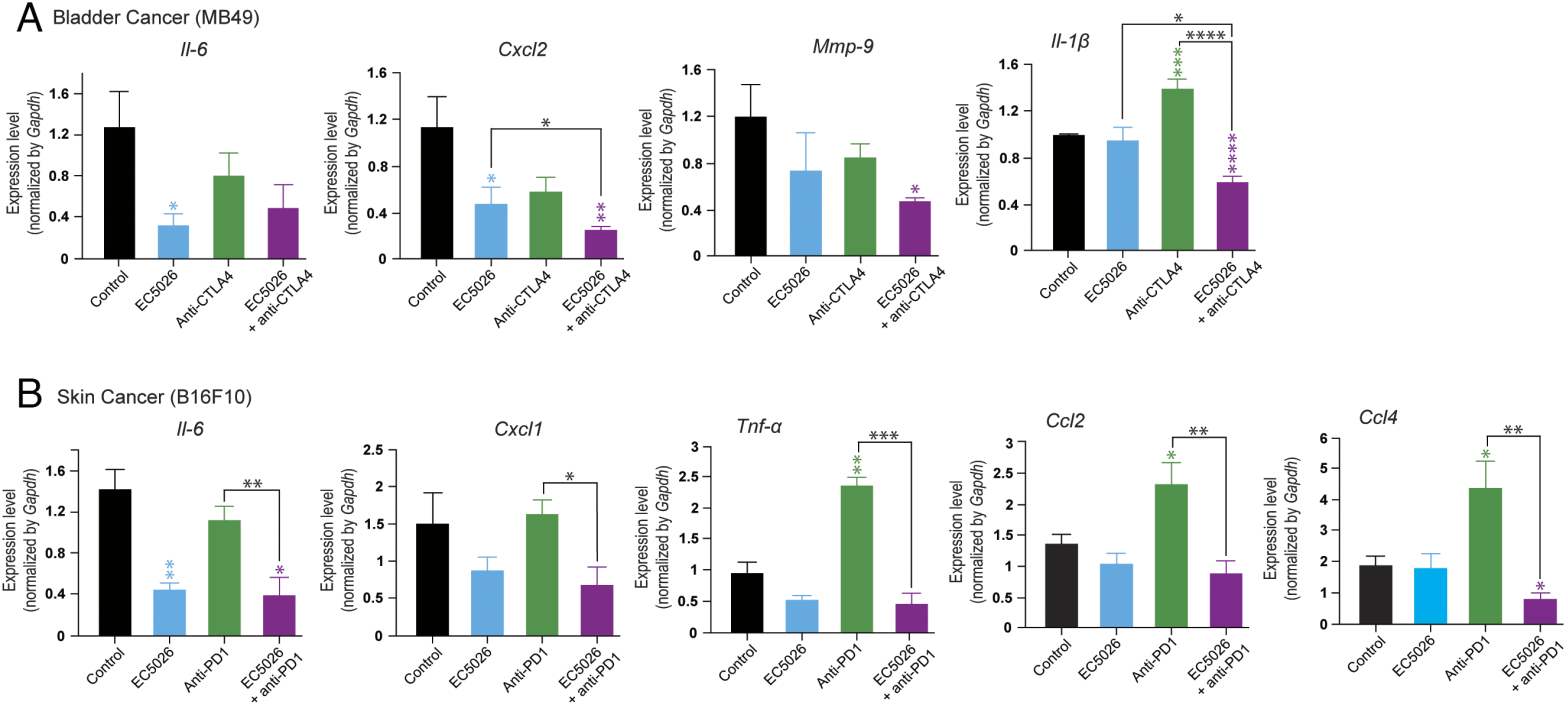
Dual ICI/sEH inhibition counters ICI-induced pro-inflammatory cytokine/chemokine gene expression. (*A*) *Il-6, Cxcl2, Mmp-9,* and *Il-1β* gene expression quantified by qPCR of tumor tissue harvested from MB49 tumor-bearing mice. (*B*) *Il-6, Cxcl1, Tnf-α, Ccl2,* and *Ccl4* gene expression quantified by qPCR of tumor tissue harvested from B16F10 tumor-bearing mice. For both (*A*) and (*B*), mice were subcutaneously inoculated with 1 × 10_6_ tumor cells (MB49 or B16F10). Treatment was initiated with either anti-PD-1 (200 μg Q3D; “anti-PD-1”), anti-CTLA4 (200 μg first dose, then 100 μg Q3D; “anti-CTLA4”), or vehicle (“control”) once tumors reached ~200 mm^3^. n = 6 to 8 mice/group. Data are represented as mean ± SEM. *P < 0.05, **P < 0.01, ***P < 0.001, ****P < 0.0001 versus control (no bracket) or comparing two groups (indicated by bracket).

We next evaluated whether counter-regulating inflammation in the TME could partly explain the observed in vivo anti-tumor activity of ICI and sEH combination therapy. We performed qRT-PCR analysis on MB49 and B16F10 tumor tissues harvested from mice that were systemically treated with either EC5026, ICIs (anti-PD-1 or anti-CTLA4), a combination of EC5026 and ICIs, or vehicle as detailed above. After 11 d of treatment, qRT-PCR identified decreased gene expression of the pro-inflammatory cytokines *Il-6*, *Cxcl2*, *Mmp9*, and *Il-1β* in EC5026 and anti-CTLA4-treated tumors, as compared to vehicle-treated tumors (Fig. 5*A* and *SI Appendix*, Fig. S1). In marked contrast, anti-CTLA4-treated tumors exhibited significantly increased levels of *Il-1β* compared to vehicle-treated tumors (Fig. 5*A*). This effect was abrogated in the tumors of mice treated with the combination of EC5026 and anti-CTLA4. To determine whether the anti-inflammatory effects of sEH inhibition combined with ICI were unique to MB49 bladder tumors and/or the specific ICI used, we next evaluated B16F10 tumors that had been treated with either EC5026, anti-PD-1, a combination of EC5026 and anti-PD-1, or vehicle. The combination of pharmacologic sEH inhibition and immune checkpoint blockade resulted in decreased gene expression of the pro-inflammatory cytokines *Il-6*, *Cxcl1*, *Tnfα*, *Ccl2*, and *Ccl4* compared to control after 11 d of treatment in the B16F10 melanoma tumor tissue (Fig. 5*B*). Interestingly, anti-PD-1 significantly increased expression of *Tnfα*, *Ccl2*, and *Ccl4* compared to vehicle-treated tumors (Fig. 5*B*). This ICIs-induced pro-inflammatory and pro-cytokine activity was neutralized with the combination treatment of EC5026 and anti-PD-1.

The occurrence of tumor-infiltrating T cells including cytotoxic CD8^+^ T cells is associated with the anti-tumor efficacy of ICI (69–71). To determine whether sEH inhibition modulates T lymphocytes in the TME, we performed flow cytometry on established MB49 bladder tumors treated with EC5026 or ICI (anti-PD-1) for 7 d. Treatment was initiated 5 d after injection of 1 × 10^6^ MB49 bladder cancer cells. Both EC5026 and anti-PD1 each significantly increased the percentage of infiltrating T lymphocytes (CD45^+^/CD3^+^) and cytotoxic CD8^+^ T cells (CD45^+^CD3^+^CD8^+^perforin^+^) in the MB49 bladder cancer tissue compared to control (*SI Appendix*, Fig. S2).

### Oxylipin Profiles Are Altered in Mice Administered Dietary ω-3 versus ω-6 PUFAs, ICI, and sEHI.

**Fig. 6.**
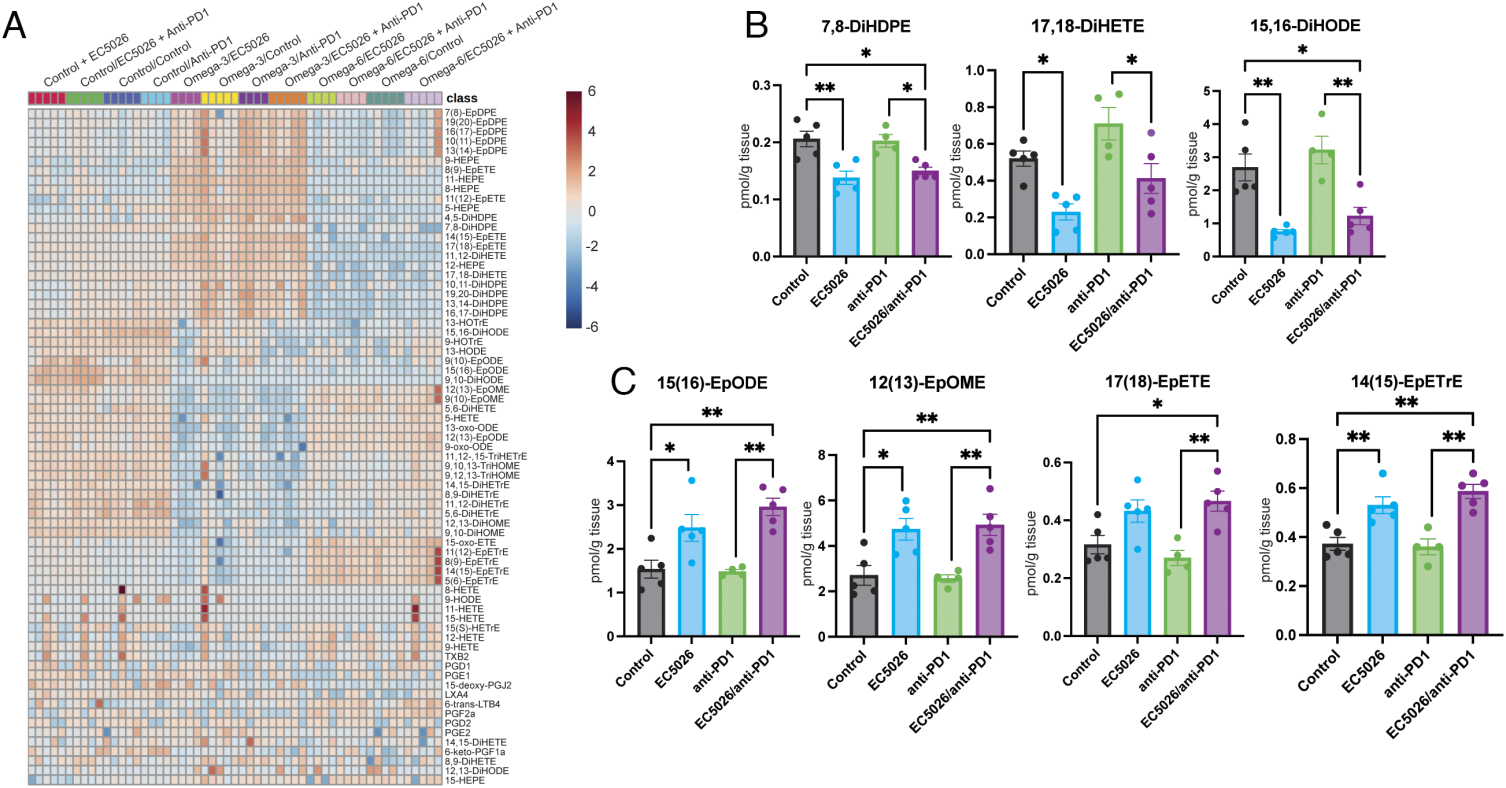
Altered oxylipin profiles in mice treated with ω-3 versus omega-6 diets, immunotherapy, and an sEH inhibitor. (*A*) Heatmap of LC-MS/MS oxylipin analysis performed on plasma from mice with bladder cancer (MB49) tumors. Plasma levels of (*B*) linoleic acid-derived oxylipins (7,8-DiHDPE, 17,18-DiHETE, and 15,16-DiHODE) and (*C*) EPA-derived oxylipins [15(16)-EpODE, 12(13)-EpOME, 17(18)-EpETE, and 14(15)-EpETrE] from tumor-bearing mice fed a control (AIN-76A) diet and treated with anti-PD1 (200 μg Q3D), EC5026 (5 mg/kg/day), a combination of anti-PD1 and EC5026, or vehicle (“control”) once tumors reached ~200 mm^3^. n = 4 to 5 mice per group. Data are represented as mean ± SEM. *P < 0.05, **P < 0.01, ***P < 0.001 for difference between two groups (groups indicated by bracket).

Based on the anti-tumor activity of ICI, dietary ω-3 PUFAs, and sEHI in our murine models, we next asked whether systemic administration of these agents can alter endogenous oxylipin levels, as quantified by UPLC-MS/MS analysis, in harvested tumor tissue and plasma. Mice were subcutaneously inoculated with 1 × 10^6^ MB49 tumor cells and randomized into diet and pharmacologic treatment groups. As expected, mice fed either an ω-3 (*SI Appendix*, Table S1) or an ω-6 PUFAs-enriched diet (*SI Appendix*, Table S2) exhibited increased levels of ω-3 PUFAs-derived or ω-6 PUFAs-derived oxylipins in harvested tumor tissues/plasma, respectively (Fig. 6*A* and *SI Appendix*, Fig. S3 and Table S3). In addition to the broad changes in oxylipin profiles induced by dietary PUFAs supplementation, treatment with systemic anti-PD-1 and EC5026 led to alterations in levels of individual metabolites as follows. Treatment with the combination EC5026 and anti-PD-1 significantly decreased the levels of 7,8-DiHDPE (ω-3 DHA-derived diol), 17,18-DiHETE (ω-6 AA-derived diol), and 15,16-DiHODE [ω-6 linoleic acid (LA)-derived diol] in plasma compared to treatment with anti-PD-1 alone (Fig. 6*B*). Likewise, treatment with both anti-PD-1 and EC5026 resulted in increased levels of 15,16-EpODE (ω-3 alpha-LA-derived epoxide), 12,13-EpOME (ω-6 LA-derived diol), 17,18-EpETE (ω-3 EPA-derived epoxide), and 14,15-EpETrE (ω-6 AA-derived epoxide) compared to anti-PD-1 treatment alone (Fig. 6*C*). These shifts in individual inflammatory oxylipins due to treatment with anti-PD-1 and sEH inhibition suggest that immunotherapy alone increases inflammation while sEH inhibition enhances the resolution of inflammatory pathways.

## Discussion

In this study, we show that treatment with anti-PD-1 induced the expression of sEH, which degrades pro-resolving EpFAs, such as EETs. Stimulating the production of endogenous pro-resolving EpFAs via ω-3 PUFAs supplementation and/or pharmacologic sEH inhibition improves the anti-tumor efficacy of ICI in murine tumor models, thus offering a molecular basis for modifying nutrition to stimulate inflammation resolution.

Like other cytotoxic cancer therapies, immunotherapy promotes inflammation within the TME, which can include cytokine release syndrome characterized by hypersecretion of pro-inflammatory cytokines, including IL-6, IL-1, TNFα, IL-5, IL-10, IFN, and TGFs by various immune cells (72). This pro-inflammatory TME, while overcoming tumor cell immune evasion, can paradoxically promote tumor growth (4). We previously demonstrated that cytotoxic cancer therapy generates robust cell death with an associated inflammatory cascade that stimulates tumor progression through increasing TME inflammation (5, 9–11, 73). Therefore, immunotherapy-induced unresolved inflammation in the TME can, in part, account for poor therapy response and relapse. Nonsteroidal and steroidal anti-inflammatory drugs that target the COX2/PGE2/EP2-4 pathway synergize with immune checkpoint blockade in mouse cancer models by remodeling the TME (69). Thus, modulation of the TME by adjuvant therapies that promotes the endogenous clearance, or resolution, of inflammation may overcome a global intrinsic limitation of immunotherapy.

As ω-3 PUFAs, such as DHA and EPA are precursors for lipid mediators of inflammation resolution, they have enormous potential as adjuvant anti-cancer therapies (4, 21, 22). For instance, dietary supplementation with ω-3 fatty acids inhibits cancer in various murine cancer models (4, 33). Preoperative ω-3 PUFAs supplementation can reduce pro-tumorigenic cytokines in cancer patients (74, 75). Consistent with these findings, our study shows that increasing the dietary ratio of ω-3:ω-6 PUFAs inhibits murine tumor growth in multiple tumor models. We also demonstrated that dietary ω-3 PUFAs supplementation enhances the efficacy of immunotherapy in preclinical murine tumor models and that anti-PD-1 potently inhibits primary tumor growth in *Fat-1* transgenic mice. Prior studies have also shown that combining an ω-3 PUFAs-enriched diet and a sEHI suppresses tumor growth by shifting the balance of fatty acid epoxides towards ω-3 metabolites (63). Here, we confirmed that an ω-3 PUFAs-enriched diet alters endogenous oxylipin levels, which may in part explain their anti-cancer activity. Thus, using both genetic and dietary methods to increase levels of ω-3 PUFAs can enhance the anti-tumor efficacy of immunotherapy.

We found that anti-PD-1 up-regulated *Ephx2*, the pro-inflammatory gene encoding sEH, in multiple tumor types. Dysregulation of *Ephx2* can contribute to carcinogenesis and more aggressive clinical phenotypes in the prostate, liver, and kidney (76–78) and is highly expressed in prostate cancer (79). Moreover, the upregulation of sEH expression has been observed in various cancers, such as seminoma, cholangiocarcinoma, and advanced ovarian cancer (80).

sEHIs are anti-inflammatory via inhibition of NF-κB and down-regulation of COX expression, as well as pro-resolving by stabilizing levels of pro-resolving EpFAs and SPMs (e.g., lipoxins and resolvins) that promote the clearance of cellular debris by local macrophages and activate anti-inflammatory cytokines (8, 28, 29, 53). Inhibition of sEH thereby may represent a unique approach to anti-cancer therapy by limiting tumor-promoting inflammation. While there are reports linking decreased sEH expression or activity to accelerated tumor growth and dissemination (40, 81), there is also clear evidence for a protective role of sEH inhibition in carcinogenesis (11, 40, 49, 58, 60, 62, 63, 82–86). For instance, sEHIs have been shown to suppress colon cancer tumorigenesis in mice (50), reduce inflammatory-driven tumor growth and metastasis in combination with COX-2 and prostaglandin receptor inhibition (9–11), and promote macrophage phagocytosis of chemotherapy-killed tumor cells which have potent pro-tumorigenic activity in vivo (2, 9–11). Interestingly, anti-cancer drugs including fulvestrant, sorafenib, and regorafenib, also inhibit sEH, which may play an underappreciated role in their anti-cancer activity (87, 88). Thus, the anti-inflammatory and pro-resolving activity of sEH inhibition by blocking pro-inflammatory cytokines may overcome the mild pro-angiogenic and growth-promoting activity of EETs (89).

Immunotherapy such as ICI can stimulate severe adverse events such as cytokine release syndrome (72, 90). In contrast to immunotherapy-induced pro-inflammatory cytokines, the sEHI alone or in combination with ICI counter-regulated pro-inflammatory cytokine production as measured by gene expression in our tumor models. The clinical significance of this may extend to cytotoxic T-cell function within the TME, which is critical for ICI efficacy. Prior studies have shown that cytokines such as IL-1β, TNFα, and IL-6/IL-6R promote inflammation that is damaging to cytotoxic T lymphocyte immunity (91–94). In our study, these cytokines were notably up-regulated by treatment with ICI alone, in contrast to treatment with EC5026 alone or in combination with ICI. In pre-clinical models, inhibition of TNFα and IL6 enhances antitumor immunity by boosting T-cell responses (19, 95). Thus, sEH inhibition may enhance immunotherapy efficacy by counter-regulating pro-inflammatory cytokine production and promoting cytotoxic T cell function in the TME. Further studies are needed to elucidate the relationship between sEH inhibition, TME inflammation, and cytotoxic T lymphocyte function.

Here, we demonstrate that dietary enrichment of ω-3 PUFAs and pharmacologic sEH inhibition enhances ICI anti-tumor efficacy in multiple murine tumor models suggesting that EpFAs may play a critical role in ICI-mediated inhibition of tumor growth. In rodent studies, sEHIs have shown promising results for controlling various inflammatory diseases, such as hypertension, atherosclerosis, diabetes, COPD, liver regeneration, fibrosis, sepsis, asthma, subarachnoid hemorrhage, arthritis, and neuropathic pain (27, 41, 57, 96–104). Thus, sEH is a critical cause of and biomarker for inflammation in many systems (40, 50, 53, 84, 99, 105–107). The human sEHI EC5026 is in clinical development as an analgesic for neuropathic pain as a nonaddictive opioid alternative (42). Another sEHI (GSK2256294) has been shown to be well tolerated in critically ill patients with subarachnoid hemorrhage, inducing an increase in serum EETs and the EET:DHET ratio (97). Moreover, the sEHI tAUCB has been shown to significantly protect against chemotherapy-induced cardiotoxicity (108). Taken together, we provide a basis for the rapid clinical translation of pharmacologic sEH inhibition as a unique and urgently needed adjuvant cancer therapy.

## Methods

### Animal Experiments and Cell Culture.

All animal studies were reviewed and approved by the Animal Care and Use Committee of Beth Israel Deaconess Medical Center. Mice were housed at a maximum of 5 mice per cage in a pathogen-free facility with unlimited access to sterile water and chow. Daily welfare evaluations and animal sacrifices were carried out according to the Committee guidelines. Six-week-old C57BL/6 mice (Jackson Laboratory, Bar Harbor, ME) were used for all tumor experiments unless indicated otherwise. RM1 (prostate cancer), MB49 (bladder cancer), and TRAMP C1 (prostate cancer) cell lines were cultured in complete medium with 10% fetal bovine serum. Adherent cells were trypsinized, pelleted, counted by hemocytometer, and resuspended in PBS. Tumor cells (1 × 10^5^ RM1 prostate tumor cells; 1 × 10^6^ tumor cells for all other cell types) were injected subcutaneously into the mid-dorsum of mice. Unless indicated otherwise, once tumors reached ~200 mm^3^, treatment was initiated with immunotherapy [anti-PD-1 (BE0146, BioXCell; a dose of 200 µg Q3D), or anti-CTLA4 (BE0164, BioXCell); for anti-CTLA4, initial dose of 200 µg then 100 µg Q3D], sEHI (TPPU or EC5026; 5 mg/kg/day via drinking water (1% PEG400 in D.I. water), or combinations thereof. Mice were killed when tumor volume reached ~2,000 mm^3^ or earlier if tumors were ulcerated.

### Diet Experiments.

Mice were fed a control diet (AIN-93G), ω-3 PUFAs-enriched diet (AIN-93G + menhaden oil), or ω-6 PUFAs-enriched diet (AIN-93G + corn oil) obtained from Research Diets (New Brunswick, NJ). For full dietary details, see *SI Appendix*, Tables S1 and S2. Twelve days following the initiation of the diet, tumor cells were injected subcutaneously into the mid-dorsum of the mice. Tramp C1 mice were killed on day 92 after initiation of the assigned diet.

## Supplementary Material

Appendix 01 (PDF)

## Data Availability

All study data are included in the article and/or *SI Appendix* and the BioStudies accession number S-BSST1321 (109).
