# Supplementary material for: Enhancing cancer immunotherapy via inhibition of soluble epoxide hydrolase
Source: Proc Natl Acad Sci U S A. 2024 Feb 8;121(7):e2314085121. doi: 10.1073/pnas.2314085121 (PMC10873624; doi:10.1073/pnas.2314085121)
Supplement: Supplementary file 1 — Appendix 01 (PDF) [file pnas.2314085121.sapp.pdf]

PNAS

Supporting Information for

Enhancing cancer immunotherapy via inhibition of soluble epoxide hydrolase

Abigail G. Kelly[a,b,c,1], Weicang Wang[d,e,1], Eva Rothenberger[a,b,c,1], Jun Yang[d,1], Molly M. Gilligan[a,b,c,1], Franciele C. Kipper[a,b,c], Ahmed Attaya[a,b,c], Allison Gartung[a,b,c], Sung Hee Hwang[d], Michael Gillespie[a,b,c], Rachel L. Bayer[a,b,c], Katherine M. Quinlivan[a,b,c], Kimberly L. Vazquez[a,b,c], Sui Huang[f], Nicholas Mitsiades[g], Haixia Yang[a,b,c,h,2], Bruce D. Hammock[d,2], Dipak Panigrahy[a,b,c,2]

Email: dpanigra@bidmc.harvard.edu

This PDF file includes:

Supporting text
Figures S1 to S4
Tables S1 to S3
Legends for Figures S1 to S4
Legends for Tables S1 to S3
SI References

Other supporting materials for this manuscript include the following:

Methods.

***Fat-1* transgenic mouse experiments.** *Fat-1* transgenic mice were obtained from Dr. Steven Freedman (Beth Israel Deaconess Medical Center, Boston, MA) and bred onsite. *Fat-1* mice were maintained on a high-ω-6 (corn oil) diet and bred heterozygote x wild type and *Fat-1* offspring confirmed via genotyping. Mice were injected with 1×10^5 RM1 prostate cells subcutaneously in the mid-dorsum. Once tumor sizes reached ~200 mm^3, treatment was initiated with anti-PD-1 (BE0146, BioXCell); 200 µL (in I.P.) (a dose of 200 mg/kg) Q3D, EC5026 (5 mg/kg/day via drinking water (1% PEG400 in D.I. water)), or both anti-PD-1 and EC5026 (both compounds prepared as aforementioned). Once tumor sizes of mice in the control group reached ~2000 mm^3, mice were sacrificed.

Gene expression analysis. RNA was extracted from tumor tissue and verified for quality and quantity using a NanoDrop Spectrophotometer (Thermo Fisher Scientific). A High-Capacity cDNA Reverse Transcription kit (Applied Biosystems) was used to reverse-transcribe isolated RNA into cDNA according to the manufacturer's instructions. A Bio-Rad CFX qPCR Instrument was used to analyze qRT-PCR with Maxima SYBR-Green Master Mix (Thermo Fisher Scientific). Target gene levels were normalized to the glyceraldehyde-3-phosphate dehydrogenase (GAPDH) gene and compared to levels in control tissues using the $2^{-\Delta\Delta Ct}$ method. The primer sequences are listed in *SI Appendix* Supplemental Fig. S3.

UPLC-MS/MS analysis. Extraction of oxylipins from tumor tissue was performed prior to UPLC-MS/MS analysis. Mice were bled retro-orbitally using micro-hematocrit capillary tubes (Fisherbrand, Pittsburgh, PA) into blood collection tubes with K2E (BD Biosciences, Billerica, MA). Whole blood was centrifuged at 10,000 rpm for 10 minutes at 4 °C within 30 minutes of collection. The supernatant was centrifuged a second time at 0.2 rcf for 5 minutes at 4 °C. The plasma layer was isolated and stored at -80 °C. Tumor tissues were collected and placed in 1 mL of methanol, gently homogenized using a glass dounce, and kept at -20 °C to allow for protein precipitation. For extracting lipid mediators from plasma or cell pellets, 200 µL of cold methanol was added to the

cell pellets in Eppendorf tubes after the addition of 10 μL of deuterated internal standards. The tubes were centrifuged for 5 min at 13,200 rpm to separate cell pellets from supernatant. The supernatants were combined with another 200 μL of ethyl acetate wash solution (to dissolve oxylipins) and dried using speed vac. The residues were reconstituted with 100 nM CUDA solution in methanol before analysis by UPLC-MS/MS. The detailed UPLC-MS/MS methods and data analysis used in this manuscript were previously reported (1, 2). The internal standard is a mixture of 500 nmol/L of nine deuterated oxylipins including: 6-keto-$PGF_{1\alpha}$-d_4, d4 PGE_2, d4 TXB_2, d4 LTB_4, d6 20 HETE, d11 14,15-DiHETrE, d8 12-HETE, d11 11,12-EpETrE. The units of the oxylipin analysis of plasma are mM (nmol/L). The speed of the centrifuge used was 16363 RCF.

Flow cytometry. Mouse MB49 bladder tumor samples were collected during surgical resection and placed in phosphate-buffered saline with 2% FBS media supplemented with 1% penicillin/streptomycin. Following the removal of fat and fibrous and necrotic areas, fresh tumor tissue specimens were cut into smaller pieces (<4 mm). Minced sample pieces were transferred to a gentleMACS C Tube (Miltenyi Biotec, catalog 130-093-237) containing 2.5 mL digestion enzyme mix prepared according to the manufacturer's instructions (Mouse Tumor Dissociation Kit, Miltenyi Biotech, catalog 130-096-730). Samples were subsequently dissociated with gentleMACS Octo Dissociator (Miltenyi Biotech) and filtered using a 70 μm nylon mesh cell strainer (Thomas Scientific, catalog 1181X53). Cells were incubated with Fixable Viability Stain (Zombie Aqua, Biolegend, catalog 423102) for 30 minutes, followed by mouse anti-Fc blocking reagent (BioLegend, catalog 101320) for 30 minutes before surface staining. Cells were stained, followed by data acquisition on the BD LSR Fortessa flow cytometer located at the Hematologic Neoplasia Core at Dana Farber Institute. Analysis was performed using FlowJo. The following antibodies were used for flow cytometry staining: CTLA-4-BV421 (clone UC10-4B9, BioLegend, 106311), Perforin-PE (clone eBioOMAK-D, ThermoFisher, 12-9392-82), CD8-BV605 (BioLegend, 100743), FoxP3-PE/eFluor 610 (clone FJK-16s, ThermoFisher, 61-5773-82), CD25-APC (clone PC61.5, BioLegend, 102012), CD45-Percp (clone 30-F11, BioLegend, 103130), PD1-APC/Cy7 (clone

29F.1A12, BioLegend, 135223), CD4-PE/Cy7 (clone RM4-5, BioLengend, 100527), and CD3-BV785 (clone 145-2C11, BioLegend, 100355).

Statistics. The statistical significance of differences between groups was analyzed by Student's two-tailed unpaired t-test between two groups and one-way ANOVA post-hoc analysis among more than two groups. P values less than 0.05 were considered statistically significant. Data are represented as mean ± SEM.

**Eurofins Sample Code:**	126-2023-10120119	**Sample Registration Date:**	12Oct2023
**Client Sample Code:**	Research Diets Modified AIN-93G w/ mostly menhaden	**Condition Upon Receipt:**	acceptable, 7.6°C
**Sample Description:**	Murine High Omega-3 Diet	**Sample Reference:**	D21121404i

QD089 - Fatty Acids-Omega 6 & 3 %W/W	Reference	Accreditation	Completed	Sub
	AOCS Ce 2-66 mod., AOCS Ce 1b-89 mod.		21Oct2023	1

Parameter	Result
Fatty Acid Profile, % Weight	Reported as Fatty Acids
C08:0 Octanoic (Caprylic)	<0.02 %
C10:0 Decanoic (Capric)	<0.02 %
Total Saturated Fatty Acids	2.13 %
C11:0 Undecanoic (Hendecanoic)	<0.02 %
C12:0 Dodecanoic (Lauric)	0.04 %
C14:0 Tetradecanoic (Myristic)	0.46 %
C14:1 Tetradecenoic (Myristoleic)	<0.02 %
C15:0 Pentadecanoic	0.04 %
C15:1 Pentadecenoic	<0.02 %
C16:0 Hexadecanoic (Palmitic)	1.29 %
C16:1 Hexadecenoic (Palmitoleic)	0.61 %
C16:2 Hexadecadienoic	0.08 %
C16:3 Hexadecatrienoic	0.05 %
C16:4 Hexadecatetraenoic	0.02 %
C17:0 Heptadecanoic (Margaric)	0.03 %
C17:1 Heptadecenoic (Margaroleic)	<0.02 %
C18:0 Octadecanoic (Stearic)	0.22 %
C18:1 Octadecenoic (Oleic + isomers)	0.86 %
C18:2 Octadecadienoic (Linoleic + isomers)	0.49 %
C18:2 Octadecadienoic Omega 6 (Linoleic)	0.45 %
C18:3 Octadecatrienoic (Linolenic + isomers)	0.06 %
C18:3 Octadecatrienoic Omega 3 (Alpha Linolenic)	0.05 %
C18:3 Octadecatrienoic Omega 6 (Gamma Linolenic)	<0.02 %
C18:4 Octadecatetraenoic Omega 3 (Stearidonic)	0.06 %
C20:0 Eicosanoic (Arachidic)	<0.02 %
C20:1 Eicosenoic (Gondoic + isomers)	0.06 %
C20:2 Eicosadienoic Omega 6	<0.02 %
C20:3 Eicosatrienoic	<0.02 %
C20:3 Eicosatrienoic Omega 3	<0.02 %
C20:3 Eicosatrienoic Omega 6	<0.02 %
C20:4 Eicosatetraenoic (Arachidonic + isomers)	0.06 %
C20:4 Eicosatetraenoic Omega 3	0.03 %
C20:4 Eicosatetraenoic Omega 6 (Arachidonic)	0.03 %
C20:5 Eicosapentaenoic Omega 3	0.27 %
C21:5 Heneicosapentaenoic Omega 3	<0.02 %
C22:0 Docosanoic (Behenic)	0.02 %
C22:1 Docosenoic (Erucic + isomers)	<0.02 %
C22:2 Docosadienoic Omega 6	<0.02 %
C22:3 Docosatrienoic, Omega 3	<0.02 %
C22:4 Docosatetraenoic Omega 6	<0.02 %
C22:5 Docosapentaenoic	0.06 %
C22:5 Docosapentaenoic Omega 3	0.05 %
C22:5 Docosapentaenoic Omega 6	<0.02 %
C22:6 Docosahexaenoic Omega 3	0.21 %
C24:0 Tetracosanoic (Lignoceric)	<0.02 %
C24:1 Tetracosenoic (Nervonic)	<0.02 %

Sum of Omega 3 Isomers	0.69 %
Sum of Omega 6 Isomers	0.52 %
Total Fatty Acids Calc.	5.10 %
Total Fat as Triglycerides	5.34 %
Total Monounsaturated Fatty Acids	1.57 %
Total Polyunsaturated Fatty Acids	1.40 %

Table S1. Composition of Omega-3 PUFA Enriched Diet (AIN-93G with menhaden oil). Fatty acids are quantified as percentages of the total diet by weight.

**Eurofins Sample Code:**	126-2023-10120120	**Sample Registration Date:**	12Oct2023
**Client Sample Code:**	Test Diet Ain-76A/10% Corn Oil 1/2 IRR	**Condition Upon Receipt:**	acceptable, 7.6°C
**Sample Description:**	Murine High Omega-6 Diet	**Sample Reference:**	Lot:23Feb22RTC1

**QD089 - Fatty Acids-Omega 6 & 3 %W/W**	**Reference**	**Accreditation**	**Completed**	**Sub**
	AOCS Ce 2-66 mod., AOCS Ce 1b-89 mod.		21Oct2023	1

**Parameter**	**Result**
Fatty Acid Profile, % Weight	Reported as Fatty Acids
C08:0 Octanoic (Caprylic)	<0.02 %
C10:0 Decanoic (Capric)	<0.02 %
Total Saturated Fatty Acids	1.71 %
C11:0 Undecanoic (Hendecanoic)	<0.02 %
C12:0 Dodecanoic (Lauric)	0.03 %
C14:0 Tetradecanoic (Myristic)	0.04 %
C14:1 Tetradecenoic (Myristoleic)	<0.02 %
C15:0 Pentadecanoic	<0.02 %
C15:1 Pentadecenoic	<0.02 %
C16:0 Hexadecanoic (Palmitic)	1.14 %
C16:1 Hexadecenoic (Palmitoleic)	0.02 %
C16:2 Hexadecadienoic	<0.02 %
C16:3 Hexadecatrienoic	<0.02 %
C16:4 Hexadecatetraenoic	<0.02 %
C17:0 Heptadecanoic (Margaric)	<0.02 %
C17:1 Heptadecenoic (Margaroleic)	<0.02 %
C18:0 Octadecanoic (Stearic)	0.39 %
C18:1 Octadecenoic (Oleic + isomers)	2.26 %
C18:2 Octadecadienoic (Linoleic + isomers)	4.55 %
C18:2 Octadecadienoic Omega 6 (Linoleic)	4.52 %
C18:3 Octadecatrienoic (Linolenic + isomers	0.58 %
C18:3 Octadecatrienoic Omega 3 (Alpha Linolenic)	0.58 %

**Parameter**	**Result**
C18:3 Octadecatrienoic Omega 6 (Gamma Linolenic)	<0.02 %
C18:4 Octadecatetraenoic Omega 3 (Stearidonic)	<0.02 %
C20:0 Eicosanoic (Arachidic)	0.03 %
C20:1 Eicosenoic (Gondoic + isomers)	0.03 %
C20:2 Eicosadienoic Omega 6	<0.02 %
C20:3 Eicosatrienoic	<0.02 %
C20:3 Eicosatrienoic Omega 3	<0.02 %
C20:3 Eicosatrienoic Omega 6	<0.02 %
C20:4 Eicosatetraenoic (Arachidonic + isomers)	<0.02 %
C20:4 Eicosatetraenoic Omega 3	<0.02 %
C20:4 Eicosatetraenoic Omega 6 (Arachidonic)	<0.02 %
C20:5 Eicosapentaenoic Omega 3	<0.02 %
C21:5 Heneicosapentaenoic Omega 3	<0.02 %
C22:0 Docosanoic (Behenic)	0.04 %
C22:1 Docosenoic (Erucic + isomers)	<0.02 %
C22:2 Docosadienoic Omega 6	<0.02 %
C22:3 Docosatrienoic, Omega 3	<0.02 %
C22:4 Docosatetraenoic Omega 6	<0.02 %
C22:5 Docosapentaenoic	<0.02 %
C22:5 Docosapentaenoic Omega 3	<0.02 %
C22:5 Docosapentaenoic Omega 6	<0.02 %
C22:6 Docosahexaenoic Omega 3	<0.02 %
C24:0 Tetracosanoic (Lignoceric)	<0.02 %
C24:1 Tetracosenoic (Nervonic)	<0.02 %

Sum of Omega 3 Isomers	0.58 %
Sum of Omega 6 Isomers	4.52 %
Total Fatty Acids Calc.	9.18 %
Total Fat as Triglycerides	9.60 %
Total Monounsaturated Fatty Acids	2.32 %
Total Polyunsaturated Fatty Acids	5.14 %

Table S2. Composition of Omega-6 PUFA Enriched Diet (AIN-93G with corn oil). Fatty acids are quantified as percentages of the total diet by weight.

Group (Diet/Treatment)	12(13)-EpODE	13-oxo-ODE	9-HOTrE	9-oxo-ODE	13-HOTrE	15(16)-EpODE	9(10)-EpODE	13-HODE	9-HODE	12(13)-EpOME	9(10)-EpOME	12,13-DiHODE	15,16-DiHODE	9,10-DiHODE	12,13-DiHOME	9,10-DiHOME	15-deoxy-PGJ2
Control/Control	0.91	0.57	0.11	3.29	0.21	1.62	0.16	4.83	N.D.	1.87	1.59	N.D.	2.12	0.15	8.85	2.93	0.23
Control/Control	0.77	0.53	0.17	2.74	0.21	1.55	0.22	3.56	N.D.	3.22	3.07	N.D.	2.11	0.22	8.44	3.19	0.29
Control/Control	0.55	0.28	0.14	1.77	0.20	1.21	0.19	4.83	0.13	2.02	1.82	N.D.	3.22	0.29	9.55	2.91	0.25
Control/Control	0.51	0.29	0.10	1.77	0.14	1.06	0.21	2.16	N.D.	2.25	2.16	N.D.	1.96	0.17	7.44	2.20	0.27
Control/Control	1.30	0.79	0.20	4.36	0.23	2.24	0.58	7.01	1.36	4.15	4.12	N.D.	4.05	0.34	13.21	3.96	0.16
Control/EC5026	0.99	0.66	0.08	3.11	0.09	1.68	0.24	0.30	N.D.	3.62	2.58	N.D.	0.77	0.24	13.21	3.59	0.31
Control/EC5026	0.79	0.46	0.07	2.35	0.10	2.42	0.32	1.78	N.D.	3.73	3.23	N.D.	0.96	0.51	13.79	4.03	0.27
Control/EC5026	1.33	0.87	0.16	4.02	0.20	3.56	0.51	5.00	0.55	5.98	3.62	N.D.	0.72	0.45	7.04	2.26	0.26
Control/EC5026	1.25	0.69	0.08	3.97	0.14	2.29	0.55	2.56	N.D.	5.59	4.42	N.D.	0.57	0.30	8.33	2.47	0.28
Control/EC5026	1.09	0.77	0.08	3.65	0.10	2.45	0.37	2.81	N.D.	4.76	3.24	N.D.	0.75	0.37	10.90	3.25	0.24
Control/Anti-PD1	0.80	0.48	0.13	2.45	0.19	1.53	0.30	3.56	N.D.	2.63	2.74	N.D.	4.31	0.31	13.17	4.67	0.30
Control/Anti-PD1	1.03	0.56	0.12	3.07	0.16	1.41	0.30	3.09	N.D.	2.92	2.64	N.D.	2.28	0.15	8.75	2.90	0.27
Control/Anti-PD1	1.25	0.70	0.19	3.93	0.24	1.39	0.26	4.84	0.21	2.58	2.36	N.D.	3.20	0.26	10.39	3.21	0.33
Control/Anti-PD1	1.38	1.04	0.19	4.29	0.21	1.59	0.23	4.93	0.52	2.11	2.00	N.D.	3.09	0.26	10.38	3.34	0.32
Control/Anti-PD1 + EC5026	1.19	0.81	0.07	4.01	0.11	2.39	0.42	2.20	N.D.	4.81	3.75	N.D.	0.73	0.37	10.17	3.50	0.26
Control/Anti-PD1 + EC5026	0.59	0.37	0.09	1.74	0.17	3.35	0.64	3.51	N.D.	5.29	4.37	0.08	2.18	0.58	12.87	2.83	0.24
Control/Anti-PD1 + EC5026	1.19	0.73	0.15	3.43	0.19	3.41	0.57	4.49	0.48	6.52	4.36	N.D.	0.96	0.62	12.89	3.76	0.20
Control/Anti-PD1 + EC5026	0.61	0.43	0.08	1.97	0.12	3.01	0.36	2.91	N.D.	4.19	3.19	0.05	1.38	0.79	14.54	3.95	0.22
Control/Anti-PD1 + EC5026	1.02	0.70	0.09	3.36	0.15	2.64	0.27	2.43	N.D.	3.82	2.42	N.D.	0.87	0.48	10.21	3.14	0.25
Omega-3/Control	0.53	0.27	0.21	2.28	0.26	1.46	1.04	20.57	7.37	3.04	3.54	N.D.	0.78	N.D.	2.18	0.53	0.25
Omega-3/Control	0.30	0.06	0.03	1.23	0.09	0.42	0.19	2.83	N.D.	0.94	0.89	1.76	0.55	N.D.	1.78	0.03	0.31
Omega-3/Control	0.41	N.D.	0.10	1.25	0.11	0.30	0.19	10.99	3.07	0.79	0.77	0.39	0.56	N.D.	1.65	0.07	0.17
Omega-3/Control	0.14	N.D.	0.01	0.58	0.02	0.15	0.08	3.74	0.01	0.82	0.72	N.D.	0.08	N.D.	N.D.	N.D.	0.21
Omega-3/Control	0.28	N.D.	0.01	0.95	0.06	0.33	0.15	2.38	N.D.	0.96	0.83	N.D.	0.70	N.D.	1.49	0.02	0.24
Omega-3/EC5026	0.31	N.D.	0.03	0.92	0.06	0.44	0.20	0.01	N.D.	1.27	0.96	N.D.	0.07	N.D.	0.53	N.D.	0.23
Omega-3/EC5026	0.12	N.D.	N.D.	0.47	N.D.	0.18	0.11	N.D.	N.D.	0.76	0.58	N.D.	0.06	N.D.	N.D.	N.D.	0.24
Omega-3/EC5026	0.20	N.D.	N.D.	0.81	0.02	0.27	0.10	0.07	N.D.	0.70	0.54	N.D.	0.04	N.D.	N.D.	N.D.	0.31
Omega-3/EC5026	0.32	N.D.	0.03	1.30	0.07	0.66	0.47	2.64	N.D.	1.73	1.57	0.16	0.05	N.D.	0.01	N.D.	0.19
Omega-3/Anti-PD1	0.37	0.03	0.00	1.51	0.06	0.65	0.42	1.69	N.D.	2.21	2.16	N.D.	0.66	N.D.	3.29	0.40	0.22
Omega-3/Anti-PD1	0.52	0.26	0.02	2.24	0.08	0.73	0.48	1.36	N.D.	1.79	1.83	0.06	0.57	N.D.	4.18	0.55	0.20
Omega-3/Anti-PD1	0.42	0.17	0.06	1.58	0.08	0.35	0.22	2.54	N.D.	1.14	0.95	N.D.	0.33	N.D.	0.75	N.D.	0.23
Omega-3/Anti-PD1	0.12	N.D.	N.D.	0.65	0.01	0.21	0.08	N.D.	N.D.	0.57	0.45	N.D.	0.26	N.D.	0.38	N.D.	0.20
Omega-3/Anti-PD1 + EC5026	0.19	N.D.	0.01	0.80	0.01	0.34	0.18	N.D.	N.D.	0.94	0.76	N.D.	0.05	N.D.	0.02	N.D.	0.20
Omega-3/Anti-PD1 + EC5026	0.30	0.03	N.D.	1.22	0.07	0.37	0.17	0.24	N.D.	0.88	0.66	0.34	0.03	N.D.	0.16	N.D.	0.30
Omega-3/Anti-PD1 + EC5026	0.15	N.D.	N.D.	0.63	0.02	0.17	0.13	N.D.	N.D.	0.67	0.62	N.D.	0.03	N.D.	N.D.	N.D.	0.22
Omega-3/Anti-PD1 + EC5026	0.23	0.00	N.D.	0.90	0.03	0.40	0.29	N.D.	N.D.	1.27	1.18	N.D.	0.05	N.D.	N.D.	N.D.	0.30
Omega-3/Anti-PD1 + EC5026	0.23	0.05	0.00	N.D.	0.04	0.59	0.33	N.D.	N.D.	1.61	1.35	0.18	0.12	N.D.	1.97	0.02	0.28
Omega-6/Control	0.97	0.39	0.02	3.47	0.03	0.34	0.21	1.21	N.D.	2.96	3.10	0.46	0.11	N.D.	0.83	N.D.	0.23
Omega-6/Control	0.80	0.39	N.D.	2.62	0.02	0.32	0.15	N.D.	N.D.	5.10	5.45	0.37	0.15	N.D.	4.49	0.77	0.19
Omega-6/Control	0.83	0.44	N.D.	3.10	0.02	0.26	0.09	N.D.	N.D.	1.73	2.10	N.D.	0.11	N.D.	2.82	0.23	0.23
Omega-6/Control	1.10	0.64	0.02	3.53	0.03	0.21	0.15	2.01	N.D.	2.98	3.59	0.15	0.14	N.D.	1.42	0.94	0.20
Omega-6/Control	1.27	0.71	0.02	3.58	0.02	0.22	0.11	0.67	N.D.	2.87	3.12	N.D.	0.29	N.D.	2.15	0.83	0.19
Omega-6/EC5026	0.97	0.62	N.D.	3.06	0.01	0.27	0.13	0.34	N.D.	3.13	3.29	N.D.	0.06	N.D.	2.02	0.56	0.20
Omega-6/EC5026	1.15	0.68	0.01	3.35	0.03	0.21	0.15	1.75	N.D.	3.06	3.49	0.55	0.05	N.D.	0.46	N.D.	0.19
Omega-6/EC5026	0.86	0.49	N.D.	2.74	0.03	0.43	0.26	0.61	N.D.	4.83	5.15	N.D.	0.09	N.D.	2.85	0.67	0.19
Omega-6/EC5026	1.08	0.57	0.04	3.47	0.03	0.12	0.12	1.44	N.D.	2.60	3.16	N.D.	0.04	N.D.	N.D.	N.D.	0.23
Omega-6/Anti-PD1	0.81	0.54	0.00	2.89	0.01	0.16	0.08	N.D.	N.D.	2.84	2.83	N.D.	0.05	N.D.	0.61	N.D.	0.18
Omega-6/Anti-PD1	1.28	0.50	0.07	3.88	0.07	0.36	0.30	4.41	0.73	3.26	3.86	N.D.	0.23	N.D.	2.32	0.45	0.20
Omega-6/Anti-PD1	1.15	0.85	0.01	3.62	0.02	0.25	0.18	1.09	N.D.	3.56	4.21	N.D.	0.13	N.D.	1.75	0.74	0.21
Omega-6/Anti-PD1	1.81	0.62	0.02	5.29	0.02	0.51	0.35	2.04	N.D.	5.60	6.48	N.D.	0.30	N.D.	6.31	1.37	0.23
Omega-6/Anti-PD1	1.45	0.88	0.03	4.19	0.03	0.26	0.18	1.66	N.D.	27.02	29.94	N.D.	0.16	N.D.	1.49	0.18	0.21
Omega-6/Anti-PD1 + EC5026	1.22	0.67	0.00	3.58	0.01	0.35	0.22	0.76	N.D.	4.01	4.07	0.05	0.03	N.D.	1.11	N.D.	0.19
Omega-6/Anti-PD1 + EC5026	1.04	0.58	0.01	2.99	0.01	0.35	0.19	N.D.	N.D.	3.96	4.05	N.D.	0.04	N.D.	1.72	0.29	0.20
Omega-6/Anti-PD1 + EC5026	0.72	0.40	N.D.	2.37	N.D.	0.18	0.09	N.D.	N.D.	3.03	2.82	N.D.	0.01	N.D.	0.80	N.D.	0.19
Omega-6/Anti-PD1 + EC5026	1.19	0.42	0.01	3.79	0.02	0.33	0.16	1.06	N.D.	4.59	4.40	N.D.	0.05	N.D.	1.24	0.45	0.20

Group (Diet/Treatment)	11-HEPE	12-HEPE	14(15)-EpETE	15-HEPE	11(12)-EpETE	15-oxo-ETE	17(18)-EpETE	5-HEPE	8(9)-EpETE	8-HEPE	9-HEPE	11(12)-EpETrE	11-HETE	12-HETE	14(15)-EpETrE	15-HETE	5(6)-EpETrE	5-HETE	8(9)-EpETrE	8-HETE	9-HETE
Control/Control	N.D.	2.16	0.18	0.09	N.D.	0.12	0.27	0.23	N.D.	N.D.	N.D.	0.63	N.D.	4.43	0.32	N.D.	2.08	0.33	0.26	N.D.	0.26
Control/Control	N.D.	1.01	0.11	0.10	0.07	0.20	0.36	N.D.	N.D.	N.D.	N.D.	0.72	N.D.	3.98	0.42	N.D.	3.28	0.30	0.37	N.D.	0.60
Control/Control	N.D.	9.00	0.08	0.09	0.12	0.18	0.26	0.09	0.41	N.D.	N.D.	0.64	0.03	70.30	0.34	0.32	3.12	0.40	0.35	0.24	4.94
Control/Control	N.D.	3.35	0.10	0.05	0.07	0.15	0.27	N.D.	N.D.	N.D.	0.65	0.63	N.D.	20.64	0.33	N.D.	2.75	0.16	0.32	N.D.	1.06
Control/Control	0.05	2.78	0.14	0.12	0.12	0.24	0.42	0.17	N.D.	N.D.	N.D.	0.82	N.D.	15.52	0.45	N.D.	4.25	0.65	0.42	N.D.	1.60
Control/EC5026	N.D.	0.72	0.12	N.D.	N.D.	0.25	0.30	N.D.	0.22	N.D.	N.D.	0.72	N.D.	5.27	0.46	N.D.	3.60	0.32	0.45	N.D.	0.76
Control/EC5026	N.D.	1.32	0.14	0.02	N.D.	0.21	0.44	N.D.	0.26	0.56	N.D.	0.81	N.D.	2.86	0.49	N.D.	3.80	0.39	0.46	N.D.	0.71
Control/EC5026	0.30	3.82	0.21	0.09	N.D.	0.34	0.54	0.00	0.19	0.60	0.95	0.96	N.D.	35.29	0.53	0.09	4.64	0.40	0.54	N.D.	3.69
Control/EC5026	N.D.	2.46	0.19	0.02	0.12	0.29	0.45	N.D.	N.D.	0.67	N.D.	1.11	N.D.	21.13	0.66	N.D.	5.24	0.25	0.56	N.D.	1.71
Control/EC5026	N.D.	0.68	0.13	0.02	N.D.	0.28	0.43	N.D.	N.D.	N.D.	N.D.	0.75	N.D.	2.60	0.51	N.D.	3.97	0.24	0.43	N.D.	0.74
Control/Anti-PD1	N.D.	1.10	0.09	0.06	0.07	0.17	0.28	0.04	N.D.	N.D.	N.D.	0.68	N.D.	3.73	0.36	N.D.	2.92	0.31	0.33	N.D.	0.72
Control/Anti-PD1	N.D.	2.30	0.14	0.07	0.03	0.23	0.34	0.10	N.D.	N.D.	N.D.	0.74	N.D.	5.04	0.44	N.D.	3.75	0.34	0.44	N.D.	0.57
Control/Anti-PD1	N.D.	1.34	0.09	0.06	0.03	0.24	0.24	0.11	0.20	0.44	N.D.	0.64	N.D.	2.26	0.36	N.D.	2.81	0.41	0.37	N.D.	0.69
Control/Anti-PD1	N.D.	1.19	0.07	0.06	0.07	0.28	0.22	0.06	0.18	N.D.	N.D.	0.59	N.D.	2.78	0.28	N.D.	2.21	0.39	0.31	N.D.	0.76
Control/Anti-PD1 + EC5026	0.12	1.61	0.17	0.01	0.06	0.31	0.46	N.D.	N.D.	N.D.	N.D.	0.94	N.D.	4.19	0.61	N.D.	4.54	0.35	0.55	N.D.	0.76
Control/Anti-PD1 + EC5026	0.63	3.75	0.19	0.06	0.10	0.14	0.57	0.06	0.25	0.88	0.93	0.90	N.D.	5.49	0.62	N.D.	4.95	0.28	0.52	N.D.	0.98
Control/Anti-PD1 + EC5026	0.22	6.68	0.18	0.06	0.03	0.27	0.50	N.D.	0.29	0.72	N.D.	1.16	N.D.	59.09	0.66	0.18	5.53	0.37	0.59	N.D.	4.09
Control/Anti-PD1 + EC5026	0.06	2.00	0.14	0.04	0.01	0.17	0.44	0.04	N.D.	0.71	0.71	0.73	N.D.	5.51	0.50	N.D.	3.65	0.22	0.42	N.D.	0.92
Control/Anti-PD1 + EC5026	0.07	2.93	0.14	0.07	0.04	0.26	0.36	0.00	N.D.	0.68	0.66	0.65	N.D.	14.51	0.54	N.D.	3.41	0.52	0.45	N.D.	1.58
Omega-3/Control	20.58	189.92	2.45	1.06	2.74	0.55	6.29	2.79	2.83	8.80	7.38	1.64	0.21	72.74	0.82	0.46	8.58	0.75	0.82	0.09	6.24
Omega-3/Control	6.06	115.91	0.54	0.38	0.59	0.13	1.92	1.90	0.75	2.07	1.89	0.38	N.D.	17.31	0.21	N.D.	1.73	0.21	0.24	N.D.	0.46
Omega-3/Control	2.43	59.09	0.30	0.22	0.22	N.D.	1.32	1.78	N.D.	N.D.	N.D.	0.26	N.D.	25.90	0.12	N.D.	0.89	0.13	N.D.	N.D.	0.88
Omega-3/Control	1.52	5.25	0.28	0.04	0.27	0.06	0.83	0.50	0.49	1.31	1.30	0.34	N.D.	1.34	0.17	N.D.	0.84	0.03	0.19	N.D.	0.19
Omega-3/Control	3.98	57.93	0.44	0.19	0.48	0.13	1.55	1.64	0.62	2.28	2.01	0.38	N.D.	13.24	0.21	N.D.	1.80	0.14	0.25	N.D.	0.33
Omega-3/EC5026	6.25	43.08	0.68	0.23	0.67	0.13	2.25	0.99	1.06	2.77	2.20	0.55	N.D.	5.87	0.33	N.D.	2.73	0.03	0.32	N.D.	0.17
Omega-3/EC5026	4.46	21.23	0.64	0.13	0.64	0.11	1.72	0.72	0.86	2.47	2.79	0.40	N.D.	5.07	0.24	N.D.	1.45	N.D.	0.23	N.D.	0.12
Omega-3/EC5026	5.13	23.22	0.58	0.14	0.55	0.15	1.88	1.29	0.75	2.48	2.30	0.39	N.D.	5.09	0.24	N.D.	1.77	0.04	0.25	N.D.	0.06
Omega-3/EC5026	10.10	51.15	0.99	0.27	1.19	0.14	3.41	1.87	1.36	4.22	4.01	0.57	N.D.	6.77	0.38	N.D.	2.83	0.13	0.35	N.D.	0.16
Omega-3/Anti-PD1	8.49	60.58	0.80	0.19	0.91	0.15	2.90	1.42	1.34	2.83	2.92	0.53	N.D.	10.14	0.36	N.D.	3.00	0.06	0.34	N.D.	0.25
Omega-3/Anti-PD1	13.20	98.03	1.07	0.21	1.27	0.28	3.72	2.15	1.21	4.52	4.01	0.66	N.D.	13.90	0.31	N.D.	2.71	0.15	0.35	N.D.	0.36
Omega-3/Anti-PD1	5.54	42.33	0.76	0.40	0.80	0.22	1.70	1.72	1.15	3.18	2.89	0.59	N.D.	8.88	0.29	N.D.	2.95	0.25	0.32	N.D.	1.23
Omega-3/Anti-PD1	5.36	24.12	0.99	0.09	0.93	0.12	2.44	1.59	1.03	3.36	3.31	0.44	N.D.	4.58	0.26	N.D.	2.13	0.10	0.30	N.D.	0.06
Omega-3/Anti-PD1 + EC5026	8.65	37.99	1.00	0.18	1.06	0.16	2.78	1.30	1.32	3.75	4.19	0.56	N.D.	7.69	0.32	N.D.	2.98	0.04	0.34	N.D.	0.19
Omega-3/Anti-PD1 + EC5026	6.28	60.98	0.62	0.13	0.60	0.20	2.16	1.23	0.77	2.51	2.26	0.34	N.D.	12.17	0.26	N.D.	1.72	0.06	0.27	N.D.	0.30
Omega-3/Anti-PD1 + EC5026	6.68	22.94	0.85	0.20	0.98	0.12	2.17	0.90	1.06	3.24	3.69	0.12	N.D.	11.69	0.29	N.D.	2.45	N.D.	0.33	N.D.	0.53
Omega-3/Anti-PD1 + EC5026	11.62	66.55	1.41	0.21	1.50	0.15	3.65	1.34	1.86	5.33	5.35	0.76	N.D.	13.11	0.43	N.D.	4.24	0.04	0.44	N.D.	0.27
Omega-3/Anti-PD1 + EC5026	15.24	72.56	1.11	0.27	1.27	0.15	4.25	1.17	1.55	4.96	4.72	0.66	N.D.	9.91	0.38	N.D.	3.68	0.04	0.39	N.D.	0.21
Omega-6/Control	N.D.	0.18	0.04	0.24	N.D.	0.46	N.D.	N.D.	N.D.	N.D.	N.D.	1.62	N.D.	27.26	0.83	N.D.	10.00	0.17	0.77	N.D.	2.69
Omega-6/Control	N.D.	0.03	0.07	0.37	0.04	0.36	N.D.	N.D.	N.D.	N.D.	N.D.	2.62	N.D.	11.47	1.30	N.D.	14.66	0.27	1.17	N.D.	0.90
Omega-6/Control	N.D.	0.30	N.D.	0.42	N.D.	0.53	N.D.	N.D.	N.D.	N.D.	0.73	1.37	N.D.	4.83	0.75	N.D.	8.14	0.20	0.66	N.D.	0.59
Omega-6/Control	N.D.	N.D.	0.06	0.33	0.02	0.38	N.D.	N.D.	N.D.	N.D.	0.77	1.37	N.D.	4.55	0.74	N.D.	7.99	0.14	0.64	N.D.	0.84
Omega-6/Control	N.D.	N.D.	0.03	0.09	0.04	0.41	N.D.	N.D.	N.D.	N.D.	0.76	1.35	N.D.	17.84	0.68	N.D.	7.95	0.29	0.66	N.D.	1.75
Omega-6/EC5026	N.D.	0.98	0.06	N.D.	0.01	0.46	0.12	N.D.	0.21	N.D.	N.D.	1.61	N.D.	23.19	0.90	N.D.	10.01	0.23	0.81	N.D.	2.46
Omega-6/EC5026	N.D.	N.D.	0.04	0.46	N.D.	0.58	N.D.	N.D.	N.D.	N.D.	N.D.	2.16	N.D.	16.49	1.15	N.D.	12.58	0.27	0.98	N.D.	1.98
Omega-6/EC5026	N.D.	1.34	0.09	N.D.	N.D.	0.27	0.17	N.D.	0.23	0.59	N.D.	1.83	N.D.	24.14	1.06	N.D.	9.95	0.24	0.82	N.D.	2.75
Omega-6/EC5026	N.D.	1.45	N.D.	0.49	0.08	0.60	N.D.	N.D.	N.D.	N.D.	N.D.	1.95	N.D.	57.72	1.06	N.D.	11.39	0.24	0.93	N.D.	4.20
Omega-6/Anti-PD1	N.D.	0.28	0.08	0.31	0.04	0.32	0.07	N.D.	0.24	N.D.	0.80	1.63	N.D.	6.51	0.88	N.D.	9.85	0.38	0.75	N.D.	0.67
Omega-6/Anti-PD1	N.D.	0.50	0.04	N.D.	0.07	0.57	N.D.	N.D.	0.28	N.D.	1.09	2.19	0.28	43.83	1.11	0.63	13.22	0.54	0.92	N.D.	6.50
Omega-6/Anti-PD1	N.D.	0.01	0.04	0.32	0.01	0.50	N.D.	N.D.	0.20	N.D.	0.82	1.98	N.D.	3.83	1.07	N.D.	11.04	0.32	0.85	N.D.	1.03
Omega-6/Anti-PD1	N.D.	0.24	0.05	0.49	0.03	0.72	N.D.	N.D.	N.D.	N.D.	0.74	3.05	N.D.	22.20	1.66	N.D.	17.90	0.42	1.31	N.D.	2.10
Omega-6/Anti-PD1	N.D.	0.16	0.23	0.48	0.36	0.76	N.D.	N.D.	N.D.	N.D.	0.73	19.59	N.D.	19.51	9.33	N.D.	113.73	0.38	8.00	N.D.	1.92
Omega-6/Anti-PD1 + EC5026	N.D.	N.D.	0.04	0.41	0.08	0.51	N.D.	N.D.	N.D.	N.D.	N.D.	2.29	N.D.	6.22	1.40	N.D.	14.45	0.37	1.11	N.D.	0.85
Omega-6/Anti-PD1 + EC5026	N.D.	0.04	0.04	0.42	0.05	0.40	N.D.	N.D.	N.D.	N.D.	0.91	1.79	N.D.	2.13	1.06	N.D.	10.70	0.29	0.81	N.D.	0.71
Omega-6/Anti-PD1 + EC5026	N.D.	N.D.	0.04	0.33	0.04	0.30	N.D.	N.D.	0.30	N.D.	0.70	1.82	N.D.	27.40	1.08	N.D.	10.01	0.18	0.80	N.D.	1.05
Omega-6/Anti-PD1 + EC5026	N.D.	0.00	0.03	0.48	N.D.	0.48	0.12	N.D.	N.D.	N.D.	0.56	1.80	N.D.	17.48	1.01	N.D.	9.15	0.24	0.80	N.D.	1.94

Table S3. Oxylipin analysis of plasma from MB49 tumor-bearing mice (corresponding to Fig. 6A).

Group (Diet/Treatment)	9-HETE	15(S)-HETrE	9,10,13-TriHOME	9,12,13-TriHOME	11,12-DiHETE	14,15-DiHETE	17,18-DiHETE	5,6-DiHETE	6-trans-LTB4	8,9-DiHETE	11,12-DiHETrE	14,15-DiHETrE	5,6-DiHETrE	8,9-DiHETrE	10(11)-EpDPE	13(14)-EpDPE	16(17)-EpDPE	19(20)-EpDPE	7(8)-EpDPE	LXA4	PGD2	PGE2	11,12-,15-TriHETrE	PGD1	PGE1	PGF2a	10,11-DiHDPE	13,14-DiHDPE	16,17-DiHDPE	19,20-DiHDPE	4,5-DiHDPE	7,8-DiHDPE	TXB2	6-keto-PGF1a
Control/Control	0.26	0.04	36.71	66.02	0.02	0.20	0.54	N.D.	0.09	0.18	0.13	0.66	0.23	0.21	1.09	0.49	0.40	2.12	5.77	99.92	5.45	N.D.	29.32	N.D.	6.79	0.43	0.08	0.08	0.14	1.09	0.23	0.24	0.34	0.33
Control/Control	0.60	N.D.	32.15	53.46	0.02	0.04	0.52	0.54	0.11	0.11	0.12	0.80	0.19	0.19	1.11	0.58	0.54	1.60	10.93	60.51	5.27	0.31	24.51	0.71	N.D.	0.75	0.06	0.08	0.15	1.03	0.08	0.21	0.48	0.15
Control/Control	4.94	0.06	33.34	57.82	0.02	0.09	0.62	0.26	0.13	0.20	0.11	0.90	0.19	0.19	0.53	0.36	0.32	0.92	2.46	332.50	5.20	0.33	27.45	5.91	11.81	2.25	0.06	0.07	0.12	1.04	0.16	0.18	4.73	0.12
Control/Control	1.06	N.D.	17.01	29.63	0.01	0.08	0.37	0.50	0.17	0.14	0.10	0.70	0.17	0.15	1.01	0.55	0.53	1.18	7.48	344.62	5.69	0.37	14.54	N.D.	N.D.	0.56	0.05	0.06	0.10	0.73	0.07	0.17	0.96	0.29
Control/Control	1.60	0.05	39.29	68.21	0.02	0.11	0.55	0.41	0.16	0.29	0.14	0.86	0.22	0.22	1.71	0.96	0.83	2.43	16.71	161.94	8.16	1.02	28.67	N.D.	N.D.	0.80	0.08	0.10	0.18	1.64	0.35	0.23	0.90	0.19
Control/EC5026	0.76	N.D.	27.61	45.58	0.02	0.06	0.32	0.34	0.11	0.18	0.13	0.94	0.21	0.23	1.02	0.64	0.55	1.40	9.07	81.00	5.39	0.29	19.81	3.30	6.42	N.D.	0.05	0.06	0.11	0.65	0.16	0.16	0.53	0.24
Control/EC5026	0.71	N.D.	29.06	45.98	0.03	0.07	0.31	0.32	0.41	0.30	0.12	1.06	0.19	0.21	1.07	0.65	0.57	1.90	9.69	282.66	4.98	0.34	21.80	2.17	6.21	0.97	0.05	0.07	0.11	0.83	0.19	0.17	0.36	0.10
Control/EC5026	3.69	0.03	33.64	57.80	0.02	0.06	0.27	0.11	0.22	0.14	0.11	0.74	0.17	0.16	1.14	0.65	0.65	1.47	9.50	835.78	5.84	0.34	22.60	9.66	8.68	N.D.	0.05	0.06	0.09	0.49	0.15	0.13	2.80	0.07
Control/EC5026	1.71	0.01	32.81	55.97	0.02	0.17	0.13	0.19	0.25	0.17	0.10	0.52	0.17	0.14	1.46	0.84	0.77	1.92	15.24	135.71	N.D.	0.35	24.14	7.10	8.17	N.D.	0.05	0.05	0.07	0.56	0.19	0.11	0.93	0.13
Control/EC5026	0.74	N.D.	27.67	52.41	0.02	0.03	0.12	0.20	0.11	0.24	0.10	0.77	0.17	0.16	0.82	0.55	0.50	1.36	6.19	46.09	N.D.	0.60	21.26	N.D.	6.29	0.53	0.05	0.05	0.08	0.67	0.16	0.12	0.51	0.07
Control/Anti-PD1	0.72	0.01	33.05	55.14	0.03	0.08	0.87	0.21	0.18	0.16	0.14	1.13	0.20	0.21	0.96	0.69	0.50	1.61	9.03	189.06	5.93	0.40	23.43	2.84	8.33	N.D.	0.07	0.08	0.16	1.44	0.17	0.18	0.62	0.16
Control/Anti-PD1	0.57	0.03	28.05	48.71	0.01	0.12	0.53	0.26	0.20	0.14	0.12	0.76	0.21	0.22	1.05	0.60	0.51	1.62	10.04	231.95	N.D.	0.38	21.40	N.D.	4.55	0.95	0.07	0.07	0.12	1.06	0.17	0.21	0.53	0.34
Control/Anti-PD1	0.69	0.02	28.29	48.56	0.02	0.10	0.85	0.17	0.09	0.17	0.14	0.93	0.22	0.24	0.74	0.59	0.47	1.14	4.19	645.80	N.D.	0.38	20.03	3.68	11.47	0.54	0.06	0.08	0.16	1.08	0.18	0.23	0.38	0.35
Control/Anti-PD1	0.76	0.02	29.76	49.19	0.03	0.06	0.59	0.30	0.13	0.19	0.13	0.87	0.23	0.24	0.56	0.34	0.30	0.94	2.62	266.62	5.49	0.35	21.78	4.75	5.55	0.99	0.06	0.07	0.14	0.85	0.20	0.19	0.47	0.34
Control/Anti-PD1 + EC5026	0.76	N.D.	29.15	46.21	0.02	0.06	0.22	0.09	0.19	0.18	0.11	0.83	0.17	0.19	1.23	0.74	0.72	1.81	12.41	141.19	5.24	0.31	19.35	N.D.	11.19	1.03	0.05	0.06	0.10	0.79	0.17	0.14	0.41	0.12
Control/Anti-PD1 + EC5026	0.98	0.02	36.36	59.19	0.05	0.08	0.66	0.07	0.20	0.28	0.12	1.67	0.20	0.20	1.64	0.90	0.85	2.18	17.73	246.31	4.93	0.30	24.37	5.84	6.03	0.61	0.07	0.08	0.15	1.63	0.20	0.16	0.62	0.12
Control/Anti-PD1 + EC5026	4.09	0.05	33.86	55.51	0.02	0.09	0.29	0.27	0.23	0.27	0.12	1.19	0.17	0.17	1.53	0.87	0.80	2.08	14.97	225.03	5.13	0.33	21.58	8.18	12.98	N.D.	0.05	0.07	0.11	0.97	0.15	0.14	2.64	0.15
Control/Anti-PD1 + EC5026	0.92	0.01	26.94	43.53	0.04	0.04	0.53	0.22	0.09	0.28	0.12	1.75	0.18	0.17	1.09	0.72	0.62	2.12	7.84	347.87	N.D.	0.42	20.63	1.81	N.D.	1.28	0.07	0.07	0.13	1.48	0.26	0.14	0.56	0.06
Control/Anti-PD1 + EC5026	1.58	0.05	36.98	61.60	0.02	0.09	0.36	0.03	0.81	0.18	0.12	1.04	0.20	0.21	0.86	0.61	0.56	1.76	7.49	121.14	5.17	0.54	23.56	5.53	5.85	0.82	0.05	0.07	0.11	1.04	0.19	0.17	1.32	0.35
Omega-3/Control	6.24	0.08	142.39	250.44	0.15	0.30	1.70	N.D.	0.11	0.31	0.10	0.36	0.18	0.14	11.23	6.11	5.60	9.57	170.60	43.52	N.D.	0.42	35.85	3.37	7.69	N.D.	0.15	0.15	0.32	3.12	0.54	0.32	2.16	0.09
Omega-3/Control	0.46	0.02	14.32	24.17	0.14	0.07	2.15	N.D.	0.09	0.30	0.09	0.42	0.15	0.12	1.97	1.25	1.27	3.34	27.19	158.38	5.19	0.29	6.93	0.85	10.26	1.27	0.10	0.11	0.23	3.22	0.65	0.31	0.52	0.07
Omega-3/Control	0.88	0.02	5.86	7.98	0.07	0.09	1.04	N.D.	0.23	0.16	0.08	N.D.	0.12	N.D.	1.25	0.66	0.93	2.45	N.D.	N.D.	6.06	0.57	N.D.	1.29	6.04	N.D.	0.14	0.08	0.21	2.25	0.50	0.20	0.42	0.11
Omega-3/Control	0.19	N.D.	19.35	43.72	0.02	0.15	0.47	N.D.	0.18	0.12	0.07	0.09	0.13	0.10	1.50	0.94	0.85	1.91	14.94	555.70	5.64	0.85	4.78	6.92	13.19	1.70	0.05	0.06	0.09	1.21	0.23	0.21	0.49	0.07
Omega-3/Control	0.33	0.00	15.85	26.78	0.11	0.06	1.66	N.D.	0.11	0.27	0.09	0.41	0.14	0.11	1.61	1.06	0.88	2.59	18.92	292.66	5.06	0.42	4.87	4.05	10.23	N.D.	0.08	0.09	0.15	2.24	0.48	0.24	0.70	0.09
Omega-3/EC5026	0.17	N.D.	6.78	13.16	0.12	0.10	0.70	N.D.	0.19	0.20	0.08	0.29	0.11	0.09	2.48	1.51	1.40	3.83	32.37	197.48	5.70	0.49	0.48	N.D.	10.12	0.29	0.08	0.09	0.11	1.98	0.56	0.16	0.37	0.06
Omega-3/EC5026	0.12	N.D.	5.97	9.67	0.07	0.08	0.30	N.D.	0.21	0.14	0.07	0.09	0.11	0.06	1.71	0.93	1.01	2.33	20.22	134.85	5.46	0.35	2.19	2.36	9.27	N.D.	0.05	0.06	0.08	0.96	0.36	0.13	0.34	0.05
Omega-3/EC5026	0.06	N.D.	5.46	11.90	0.09	0.09	0.51	N.D.	0.15	0.17	0.07	0.07	0.12	0.08	1.64	0.92	0.95	2.58	18.39	171.31	5.27	0.34	1.25	1.66	6.23	1.54	0.06	0.07	0.12	1.30	0.36	0.14	0.37	0.06
Omega-3/EC5026	0.16	0.04	6.72	10.13	0.09	0.15	0.69	N.D.	0.20	0.19	0.08	N.D.	0.12	0.09	3.61	2.00	2.10	5.28	50.61	186.59	6.19	0.73	1.50	2.32	4.40	N.D.	0.08	0.10	0.21	1.82	0.61	0.20	0.46	0.08
Omega-3/Anti-PD1	0.25	0.00	6.19	13.62	0.14	0.06	1.96	0.02	0.15	0.31	0.09	0.26	0.11	0.11	4.32	2.52	2.49	7.86	55.89	110.19	5.36	0.72	1.59	N.D.	N.D.	N.D.	0.11	0.11	0.29	4.99	0.55	0.30	0.49	0.08
Omega-3/Anti-PD1	0.36	0.02	3.61	9.94	0.15	0.50	2.29	N.D.	0.22	0.27	0.10	0.21	0.12	0.10	4.17	2.48	2.28	6.45	59.33	N.D.	N.D.	0.49	1.94	N.D.	N.D.	1.71	0.13	0.13	0.35	5.41	0.97	0.25	0.37	0.23
Omega-3/Anti-PD1	1.23	0.03	11.86	20.58	0.09	0.13	1.20	0.02	0.15	0.22	0.07	0.51	0.12	0.08	3.91	2.29	2.06	4.72	53.93	589.62	5.43	0.44	6.94	5.74	7.78	0.83	0.08	0.09	0.16	2.44	0.33	0.20	0.64	0.06
Omega-3/Anti-PD1	0.06	N.D.	4.94	11.38	0.14	0.10	1.89	N.D.	0.20	0.29	0.08	0.29	0.16	0.12	2.23	1.14	1.22	3.75	26.17	118.03	5.21	0.31	3.08	0.89	N.D.	N.D.	0.09	0.09	0.21	2.66	0.47	0.29	0.39	0.04
Omega-3/Anti-PD1 + EC5026	0.19	0.00	5.44	11.59	0.11	0.09	0.82	N.D.	0.14	0.21	0.07	0.08	0.11	0.08	3.65	1.94	1.96	5.15	45.21	180.65	5.32	0.45	1.26	2.46	9.91	N.D.	0.07	0.09	0.15	2.40	0.66	0.18	0.45	0.04
Omega-3/Anti-PD1 + EC5026	0.30	N.D.	5.19	7.20	0.09	0.05	0.78	N.D.	0.24	0.20	0.08	0.02	0.12	0.09	1.86	1.01	1.15	3.81	22.92	85.59	5.67	0.47	N.D.	N.D.	4.20	N.D.	0.14	0.09	0.17	2.04	0.81	0.18	0.50	0.26
Omega-3/Anti-PD1 + EC5026	0.53	N.D.	5.16	11.46	0.03	0.12	0.29	N.D.	0.26	0.12	0.06	N.D.	0.11	0.06	2.25	1.39	1.35	2.81	27.17	85.91	5.32	0.29	2.72	1.11	4.90	N.D.	0.04	0.05	0.08	0.97	0.27	0.12	0.52	0.04
Omega-3/Anti-PD1 + EC5026	0.27	0.01	2.53	7.26	0.12	0.10	0.67	0.06	0.20	0.23	0.08	0.12	0.12	0.08	4.51	2.38	2.36	7.13	58.81	100.59	5.02	0.40	0.07	N.D.	N.D.	0.90	0.08	0.08	0.14	2.04	0.80	0.15	0.48	0.02
Omega-3/Anti-PD1 + EC5026	0.21	0.02	5.15	10.41	0.24	0.04	1.63	N.D.	0.12	0.29	0.10	0.37	0.12	0.12	4.40	2.62	2.61	7.05	61.29	7.02	N.D.	0.27	1.27	1.67	N.D.	N.D.	0.11	0.14	0.30	4.03	0.96	0.20	0.41	0.24
Omega-6/Control	2.69	0.02	11.32	21.40	N.D.	0.10	N.D.	0.32	0.25	0.05	0.07	0.23	0.13	0.11	0.66	0.41	0.39	0.58	5.35	N.D.	5.17	0.28	5.37	N.D.	5.81	0.49	0.07	0.03	0.03	0.24	0.07	0.09	1.26	0.03
Omega-6/Control	0.90	0.00	9.84	19.81	N.D.	0.14	0.05	0.15	0.13	0.32	0.11	0.67	0.16	0.15	1.42	0.78	0.67	1.65	15.53	49.37	N.D.	N.D.	2.71	N.D.	N.D.	N.D.	0.04	0.04	0.08	0.90	0.13	0.12	0.65	0.12
Omega-6/Control	0.59	N.D.	9.82	18.73	N.D.	0.06	0.01	0.10	0.17	0.09	0.10	0.62	0.15	0.14	0.51	0.29	0.29	0.55	1.95	N.D.	N.D.	0.45	2.83	2.83	4.31	1.29	0.05	0.03	0.06	0.51	0.20	0.09	0.38	0.02
Omega-6/Control	0.84	N.D.	16.34	33.86	N.D.	0.07	N.D.	0.06	0.17	0.10	0.09	0.43	0.15	0.15	0.50	0.28	0.27	0.51	1.40	N.D.	5.42	0.69	11.12	N.D.	8.71	1.56	0.06	0.03	0.05	0.35	0.07	0.10	0.48	0.02
Omega-6/Control	1.75	0.01	13.27	22.39	N.D.	0.09	0.00	0.31	0.25	0.11	0.10	1.13	0.16	0.14	0.48	0.32	0.33	0.62	2.04	9.67	4.87	0.28	7.23	6.61	4.71	1.18	0.03	0.04	0.05	0.52	0.15	0.11	1.41	0.08
Omega-6/EC5026	2.46	0.01	15.96	24.99	N.D.	0.04	0.04	0.17	0.25	0.16	0.09	0.81	0.14	0.14	0.95	0.53	0.51	1.09	8.78	113.33	5.23	0.26	9.59	3.41	N.D.	1.65	0.03	0.04	0.05	0.54	0.17	0.09	1.23	0.05
Omega-6/EC5026	1.98	0.02	19.73	39.55	N.D.	0.06	N.D.	0.06	0.25	0.24	0.09	0.23	0.12	0.10	0.60	0.33	0.34	0.54	2.80	N.D.	5.75	0.51	9.94	N.D.	N.D.	2.48	0.03	0.03	0.03	0.26	0.09	N.D.	0.71	0.05
Omega-6/EC5026	2.75	0.02	17.79	29.10	N.D.	0.08	0.03	0.21	0.27	0.17	0.08	0.72	0.12	0.11	1.33	0.75	0.69	1.37	12.52	298.66	5.39	0.32	10.91	N.D.	N.D.	1.24	0.03	0.04	0.05	0.61	0.16	0.10	1.18	0.11
Omega-6/EC5026	4.20	0.03	14.49	28.23	N.D.	0.15	N.D.	0.02	0.33	0.13	0.08	0.07	0.14	0.10	1.07	0.70	0.58	0.90	11.42	N.D.	N.D.	0.31	9.62	N.D.	N.D.	N.D.	0.08	0.04	0.04	0.32	0.16	N.D.	1.41	0.16
Omega-6/Anti-PD1	0.67	0.02	13.15	22.44	N.D.	0.10	0.11	0.24	0.36	0.19	0.09	0.79	0.19	0.15	1.34	0.62	0.65	1.57	13.39	N.D.	N.D.	0.38	8.66	5.00	4.49	1.24	0.04	0.04	0.07	0.93	0.20	0.14	0.60	0.22
Omega-6/Anti-PD1	6.50	0.08	41.40	67.55	N.D.	0.09	N.D.	0.23	0.34	0.08	0.10	1.36	0.19	0.14	0.85	0.50	0.42	0.89	6.87	141.47	5.29	0.39	34.07	3.62	N.D.	2.47	0.04	0.04	0.05	0.60	0.11	0.11	3.28	0.09
Omega-6/Anti-PD1	1.03	N.D.	18.68	31.90	N.D.	0.01	N.D.	0.35	0.26	0.28	0.10	1.21	0.14	0.12	0.68	0.34	0.36	0.69	2.42	132.93	5.15	N.D.	12.31	3.96	6.91	1.63	0.03	0.03	0.05	0.50	0.10	N.D.	0.39	0.05
Omega-6/Anti-PD1	2.10	0.05	19.75	31.06	N.D.	0.06	0.06	0.22	0.41	0.21	0.12	1.36	0.18	0.18	1.32	0.65	0.67	1.46	13.15	202.62	5.38	0.87	16.33	2.27	N.D.	2.68	0.04	0.04	0.07	0.82	0.21	N.D.	0.65	0.07
Omega-6/Anti-PD1	1.92	0.01	18.56	41.23	N.D.	0.11	0.00	0.20	0.33	0.13	0.10	0.57	0.16	0.17	7.95	4.24	4.02	8.21	130.10	47.65	N.D.	0.30	14.22	N.D.	6.71	1.69	0.06	0.04	0.06	0.70	0.17	N.D.	0.65	0.28
Omega-6/Anti-PD1 + EC5026	0.85	0.01	11.98	26.20	N.D.	0.07	N.D.	0.18	0.29	0.27	0.09	0.51	0.13	0.12	0.72	0.47	0.42	0.92	5.08	N.D.	5.29	0.26	8.72	2.90	5.85	1.79	0.03	0.03	0.04	0.38	0.21	0.08	0.45	0.05
Omega-6/Anti-PD1 + EC5026	0.71	N.D.	12.42	19.46	N.D.	0.07	0.02	0.35	0.27	0.14	0.09	0.88	0.15	0.13	0.73	0.42	0.41	0.82	6.02	216.75	5.11	0.28	2.09	2.05	N.D.	0.63	0.05	0.03	0.04	0.50	0.19	0.08	0.43	0.20
Omega-6/Anti-PD1 + EC5026	1.05	0.00	10.99	19.67	N.D.	0.07	N.D.	0.25	0.29	0.19	0.09	0.77	0.14	0.12	0.72	0.39	0.38	1.00	4.52	257.87	5.42	0.33	7.45	1.61	6.42	1.30	0.03	0.03	0.04	0.47	0.19	0.08	0.80	0.21
Omega-6/Anti-PD1 + EC5026	1.94	N.D.	14.50	31.00	N.D.	0.05	N.D.	0.23	0.34	0.21	0.09	0.43	0.12	0.11	0.52	0.31	0.32	0.63	2.94	53.66	5.52	0.36	10.85	1.05	8.50	1.55	0.03	0.03	0.04	0.33	0.09	N.D.	0.64	0.05

Table S3 (cont'd). Oxylipin analysis of plasma from MB49 tumor-bearing mice (corresponding to Fig. 6A).

Gene	Sense	Antisense
Il-6	AGCCAGAGTCCTTCAGA	GGTCCTTAGCCACTCCT
Cxcl-2	GAAGACCCTGCCAAGGGTTG	AGGCAAACTTTTTGACCGCC
Mmp-9	CTTCTGGCGTGTGAGTTTCCA	ACTGCACGGTTGAAGCAAAGA
Il-1β	GCAACTGTTCCTGAACTCAACT	ATCTTTTGGGGTCCGTCAACT
Cxcl1	TCTCCGTTACTTGGGGAC	CCACACTCAAGAATGGTCGC
Tnf-α	CATCTTCTCAAAATTCGAGTGACAA	TGGGAGTAGACAAGGTACAACCC
Ccl2	ACTGAAGCCAGCTCTCTCTTCCTC	TTCCTTCTTGGGGTCAGCACAGAC
Ccl4	CCCACTTCCTGCTGTTTCTC	GAGGAGGCCTCTCCTGAAGT

Figure S1. Target gene primers used for qPCR of MB49 and B16F10 tumor tissue.

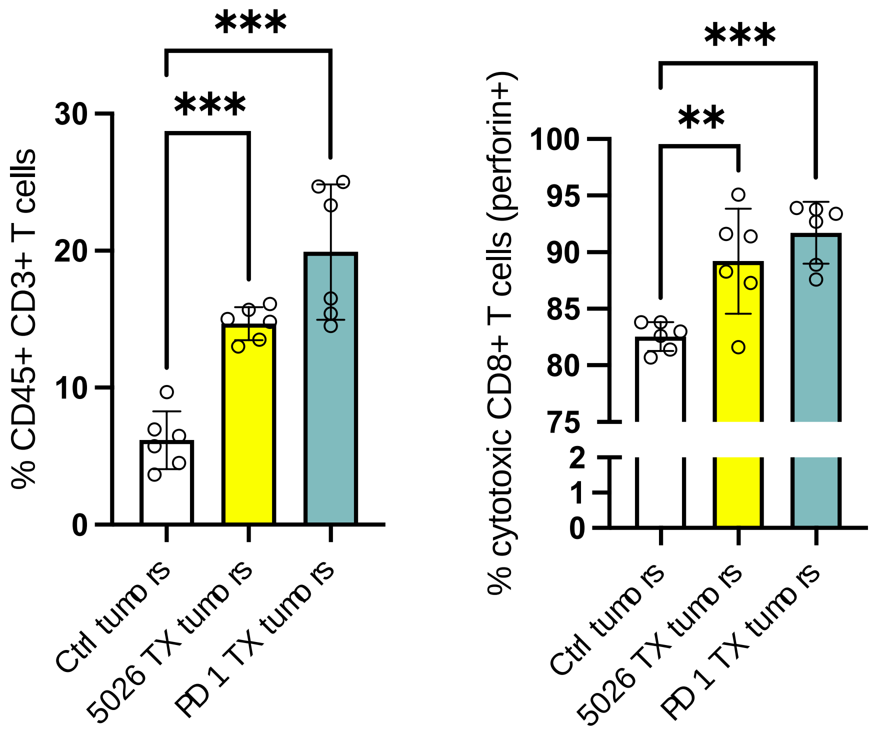

Figure S2. Anti-PD1 and EC5026 single therapy increases T cell infiltration in the tumor microenvironment. (A) T cell and (B) Cytotoxic T cel infiltration as quantified by flow cytometry in tumor tissue from MB49 tumor-bearing mice. Mice were treated with Anti-PD1, EC5026, or control as previously described once tumors reached ~200 mm^3. n = 6 mice per group.). Data are represented as mean ± SD. ***p< 0.001 by one-way ANOVA with Bonferroni's post hoc test.

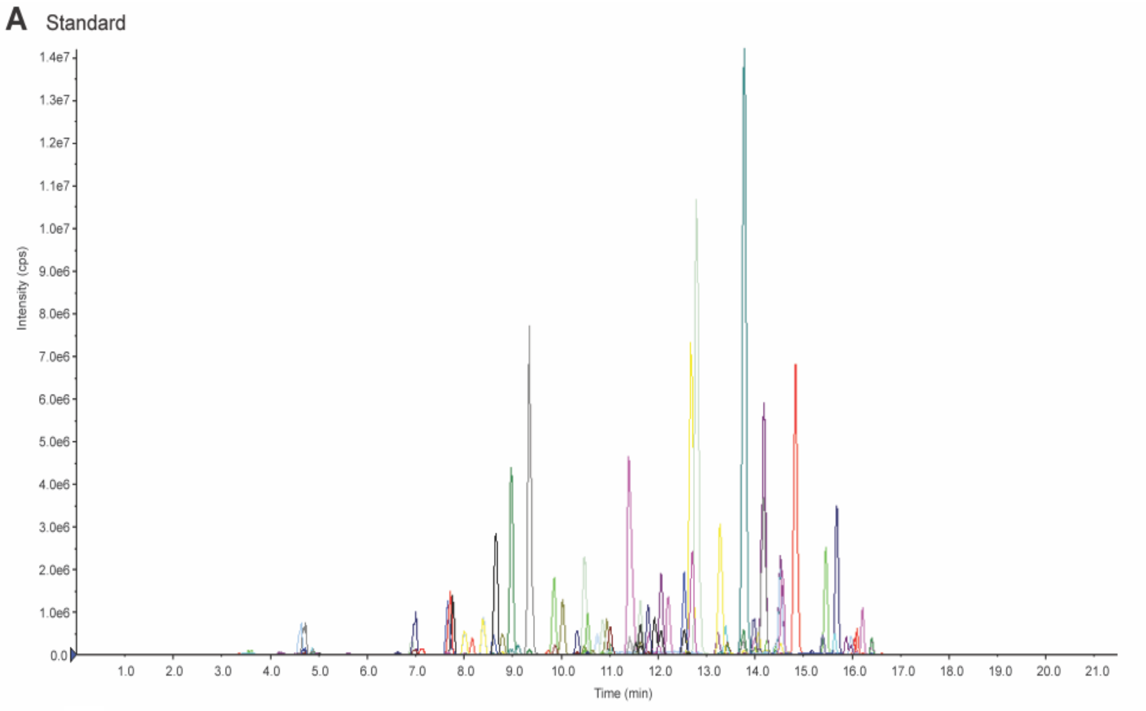

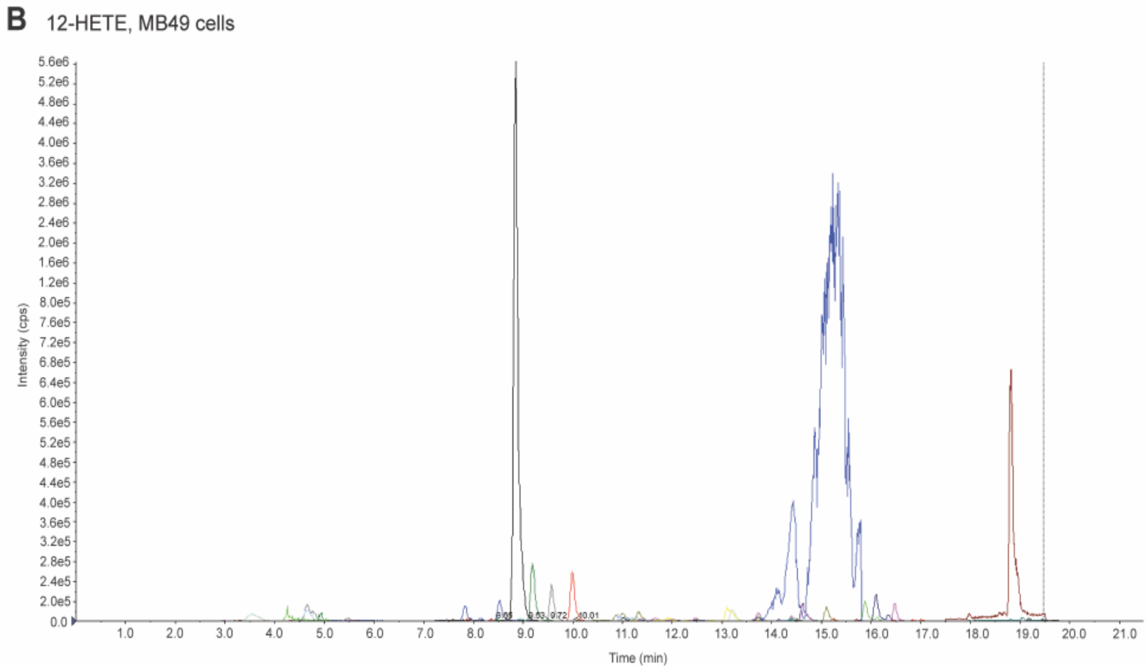

Figure S3. UPLC-MS/MS chromatogram sample for 12-HETE in plasma from MB49 tumor-bearing mice.

SI References

1. J. Yang, K. Schmelzer, K. Georgi, B. D. Hammock, Quantitative profiling method for oxylipin metabolome by liquid chromatography electrospray ionization tandem mass spectrometry. *Anal Chem* **81**, 8085-8093 (2009).
2. J. Yang *et al.*, Lipidomes of brain from rats acutely intoxicated with diisopropylfluorophosphate identifies potential therapeutic targets. *Toxicol Appl Pharmacol* **382**, 114749 (2019).
